# Impact of COVID-19 on colorectal cancer screening in a federally qualified health center: Provider and staff perspectives

**DOI:** 10.1371/journal.pone.0340184

**Published:** 2026-01-13

**Authors:** Jennifer L. Schneider, Jennifer S. Rivelli, Anne L. Escaron, Rebecca Crocker, Robert Lara, Alexis Rodriguez, Joanna Garcia, Gloria D. Coronado

**Affiliations:** 1 Kaiser Permanente Northwest Center for Health Research, Portland, Oregon, United States of America; 2 Institute for Health Equity, AltaMed Health Services Corporation, Los Angeles, California, United States of America; 3 University of Arizona, School of Public Health, Cancer Center, Tucson, Arizona, United States of America; Wayne State University, UNITED STATES OF AMERICA

## Abstract

The COVID-19 pandemic and associated policies discouraging routine-care visits dramatically affected cancer screening in the United States. Yet little is known about the perspectives of providers and staff at federally qualified health centers (FQHCs) on colorectal cancer (CRC) screening during this period. We administered hour-long telephone interviews with clinic leadership and frontline staff at a Los Angeles-based FQHC. Questions explored barriers and facilitators to CRC screening, and adaptations implemented to address challenges posed during three phases of the COVID-19 pandemic: early (March-July 2020), middle (August – December 2020), and later (January – December 2021). All interviews were recorded, transcribed, and content-analyzed by trained and experienced qualitative methodologists. Participants’ (n = 20) ages ranged from 26–56; most were female (85%), and in leadership positions (65%). Early in the pandemic CRC screening efforts were severely hampered as the health center was inundated with upper respiratory infections, lacked infrastructure to rapidly scale telehealth, and had minimal guidance from prior disaster plans geared for short-term challenges (e.g., flu). Limited availability of stool test kits and reduced gastroenterology capacity also thwarted CRC screening delivery. Throughout the pandemic, the FQHC faced challenges hiring and retaining staff for cancer screening outreach. In the later phase of the pandemic, the FQHC responded by expanding CRC screening options, deploying health record tools enabling automated outreach, and retraining staff on CRC screening workflows. Interviewees identified strategies to strengthen pandemic preparedness and sustain CRC screening, including improved monitoring of infectious disease through incoming patient call data to guide staffing decisions, diversifying vendor relationships and specialty care partnerships to ensure access, particularly in gastroenterology, and increasing home-based testing options. Our findings, while limited to the experience of one large FQHC, may help inform efforts to develop guidance that community health centers can apply in responding to future pandemics or other natural disasters to minimize impacts on CRC screening.

## Introduction

The COVID-19 pandemic and associated stay-at-home policies in the United States led to dire challenges for health care systems and dramatic interruptions to preventive care, including cancer-screening services [1–6]. Colorectal cancer (CRC) is the second leading cause of cancer death in the United States and screening can prevent cancer and find it in early, treatable stages [7]. Some studies estimate that more than half of CRC deaths in the United States could be avoided if more people were routinely screened [8]. However, at the onset of the pandemic, the American Cancer Society discouraged health care visits for routine cancer screenings, and national gastroenterology associations recommended that endoscopy units temporarily prioritize urgent procedures (i.e., patients with bleeding, obstructions, or other symptoms) and postpone all other procedures, including screening [9–11]. Additionally, the Centers for Medicare and Medicaid Services expanded reimbursement for telehealth visits, removing location restrictions and authorizing telehealth reimbursement for patients who lived in health professional shortage areas, rural census tracts, or counties outside metropolitan statistical areas [12]. This change led to a 41% nationwide increase in telehealth use from April 2019 to April 2020, partially offsetting the effects of the 68% reduction in face-to-face office visits observed during that time [13]. Nonetheless, significant disruptions to care persisted, including an estimated 95,000 missed CRC screenings from January to June 2020, contributing to elevated cancer and other disease risks [14].

Analyses of data from large surveillance systems and integrated care-delivery systems have estimated the number of missed diagnoses and excess deaths as a result of COVID-19, however these estimates were derived from a predominantly privately insured population. Our study helps give a fuller picture of the impacts of the COVID-19 pandemic on cancer screening and follow-up by studying a diverse population that receives care in Federally Qualified Health Centers (FQHCs), which serve more than 30 million individuals in the United States. Data from FQHCs nationally showed a 5.5-percentage-point drop in CRC screening (45.6% in 2019 vs. 40.1% in 2020), with a slight recovery in 2021 (41.9%), 2022 (42.8%), and 2023 (41.1%) data. Notably, in 2023, health centers began reporting CRC screening for adults 45–75 years of age because of a change to the US Preventive Services Task Force’s (USPSTF) recommendation that lowered the age to begin screening from 50 to 45 [15].

The **RESTORE (Assessing long-term impacts of the COVID-19 pandemic on disparities in cancer screening and follow-up)** study partnered with one of the largest FQHCs in the United States to examine how the COVID-19 pandemic affected CRC and other cancer screenings among populations heavily impacted by COVID in southern California. Here, we report results from a qualitative sub-study that collected data from interviews with FQHC leadership and frontline staff who had active roles in executing CRC screening and managing the FQHC’s response to the pandemic. We sought to gather in-depth contextual data about barriers and facilitators to delivering CRC prevention services across multiple time points during the pandemic. Our findings can guide health systems serving vulnerable populations in preparing for future care disruptions by applying lessons learned to improve care delivery.

## Methods

### Study setting

The study was conducted at an FQHC that operates 25 primary care clinics and provides medical care to more than 230,000 patients each year in Los Angeles County and Orange County, California (urban areas). Approximately 91% of clinic patients are classified as members of a racial/ethnic population subgroup based on medical record information, with 82% identifying as Latino. Prior to the pandemic, the FQHC had a robust CRC screening program with established workflows for both in-clinic and mailed stool-test distribution using the fecal immunochemical test (FIT), which detects blood in stool. For patients attending in-clinic visits, clinician teams determined whether they were due for CRC screening and, if due, educated patients on the importance of screening, offered the FIT, and reviewed instructions (including demonstrations) for at-home FIT completion. Clinicians offered shared decision-making to patients who desired more discussion, declined the FIT, or preferred screening via a colonoscopy. Each year for the past seven years, the FQHC also conducted a centralized mailed FIT outreach program that delivered FITs and reminders to patients due for CRC screening. Patients with abnormal FIT results received referrals for colonoscopy with a gastroenterology (GI) specialist external to the FQHC. According to Uniform Data System data, although the FQHC remained open for patient care throughout all phases of the pandemic, CRC screening rates at the FQHC dropped precipitously from 63.2% in 2019, to 42.6% in 2020, but rebounded more steeply than the national average, reaching 51.1% in 2021 [16–18].

**Fig 1:**
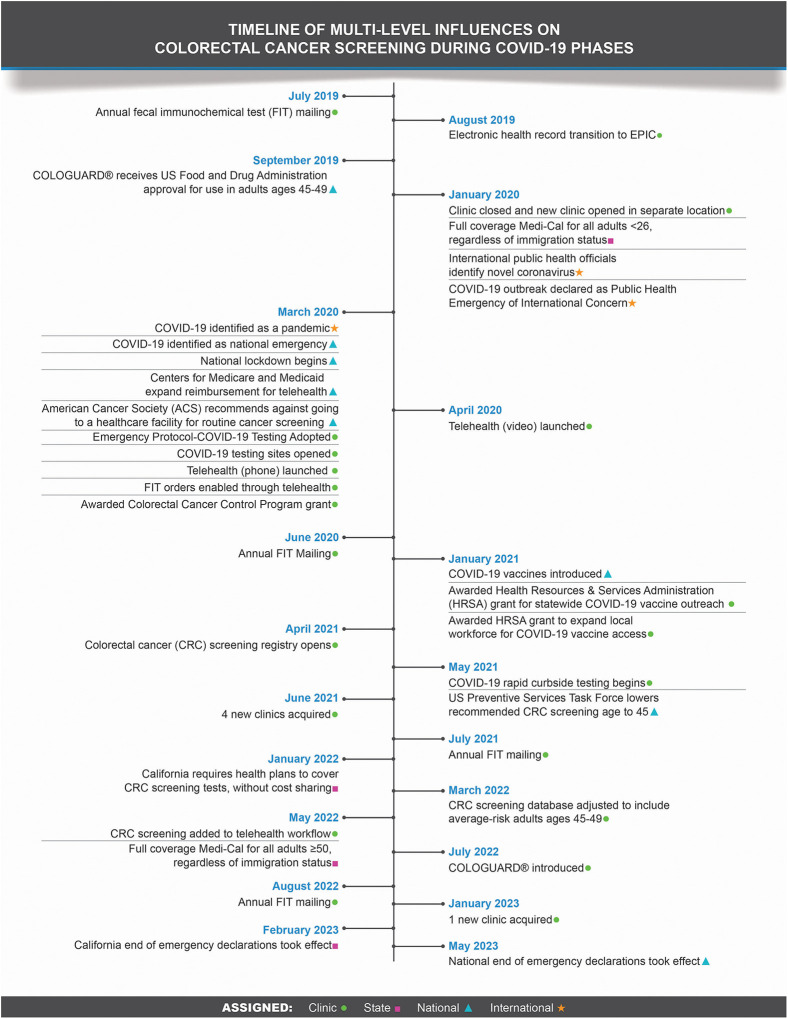
Timeline of multi-level influences on colorectal cancer screening during COVID-19 pandemic.

Multi-level state and national policy changes occurred before, during, and after the pandemic (Fig 1). The COVID-19 pandemic led to national lockdowns and CRC screening postponements beginning in March 2020. Policy changes expanded eligibility and coverage, including: the Centers for Medicare and Medicaid Services’ expanded reimbursement policy for care delivered via telehealth; the USPSTF recommendation to lower the screening age to 45 (May 2021; from the prior recommendation of 50); and California’s mandate to cover CRC screening without cost-sharing (January 2022). The end of emergency declarations took effect in February 2023 in California and May 2023 at the national level.

At the local FQHC level, multiple changes occurred throughout the pandemic period. These included: clinic transitions (clinic location closed and reopened in a separate location) and expansions (new clinics); changes in health information technology (IT) infrastructure; and new screening modalities and outreach initiatives (Fig 1). Clinic transitions occurred before and during the pandemic (February 2018, January 2020), while new clinics were established post-pandemic (June 2021, January 2023, October 2023). A transition to a new electronic health record (EHR) system (August 2019) and integration of CRC screening into telehealth workflows (May 2022) also took place during this period. Additionally, the health center introduced new CRC screening modalities, i.e., FIT-DNA testing (July 2022), and outreach initiatives, funded through external agencies (January 2021).

### Qualitative aim and design

The **RESTORE** study seeks to assess the impacts of COVID-19 care suspensions and policies on cancer preventive care delivery, specifically for breast, cervical, and CRCs, and to develop guidance for responding to disruptions and improving care delivery. Here, we present findings from qualitative interviews with clinic staff to understand COVID-19’s impact on CRC screening and follow-up.

Several members of the research team (JLS, JSR, ALE, GC) developed a semi-structured interview guide (see S1 Appendix) that included questions about cancer screening processes over distinct phases of the COVID-19 pandemic. Phases were: 1) early pandemic, characterized by “shutdown” and stay-at-home orders (March-July 2020); 2) mid pandemic, characterized by “reopening” and testing for COVID-19 (August – December 2020); and 3) late pandemic, characterized by roll-out of COVID-19 vaccines (January – December 2021). We also explored the current state of cancer screening at the FQHC at the time interviews were conducted (approximately March 2022 – January 2023). Overall, our questions explored the impact of the pandemic on clinical practices to promote cancer screening, health system and patient-level barriers and facilitators to implementing cancer screening, long term COVID-19 impacts on clinics and patients, and possible suggestions for future actions and improvements to workflows. Here, we report findings from questions focused specifically on the impact of COVID-19 on the FQHC’s CRC screening efforts.

### Data collection and recruitment

The study sample included current FQHC staff representing a range of roles pertaining to cancer screening and/or guiding organizational responses to the pandemic. These staff included medical directors, operations directors, managers, clinicians (primary care providers and nurses), and frontline patient-facing staff (medical assistants, health educators, referral coordinators, and front desk staff). Our goal was to interview approximately 20 individuals, a sample size sufficient for gathering a depth and range of experiences, based on our own experiences and the literature [19].

We used purposeful sampling techniques in which we identified an initial list of staff for interviews (n = 21) based on recommendations from study staff (ALE) employed by the FQHC [20–22]. We used a snowball-sampling technique where we asked interviewees for recommendations of additional staff to interview; this generated an additional five possible participants. Study staff employed at the FQHC sent recruitment letters (RL, JG) and a consent information sheet to potential participants via email. No incentive was offered per clinical guidelines. Master’s level trained qualitative research specialists (JLS, JSR) then scheduled telephone interviews. Interviewers reviewed the consent information sheet emailed to participants and answered any questions, then obtained verbal consent (a waiver of signed consent was approved by the IRB) prior to commencement. Interviews lasted from 45 to 60 minutes and occurred between March 2022 and January 2023. All study materials and processes for the interviews were reviewed and approved by the Kaiser Permanente Interregional Institutional Review Board (protocol # 1752845, 2/23/2022).

### Analysis

Interviews were audio-recorded with verbal consent from participants and professionally transcribed. The qualitative team (JLS, JSR) followed a rapid, iterative content-analysis approach using topical summarization techniques [23–25]. First, qualitative staff reviewed both the interview guide and a subset of transcripts to develop a topical extraction template in Microsoft Word (see S2 and S3 Appendix). The topical extraction template reflected both key areas of the interview guide (e.g., CRC screening barriers and telehealth) across pandemic time periods (e.g., early, mid, later), as well as emergent themes from transcript review. Next, the qualitative team reviewed each transcript in detail to extract data, including key feedback for each interview question along with direct quotes, relevant to the topical extraction template. During this process, JLS and JSR met regularly to discuss the extraction process and create initial summary reports. These initial summary reports were iteratively reviewed and refined through repeated review of interview transcripts, including identifying sub-topics to support main topics (e.g., vendor challenges [sub-topic] related to CRC screening during early, mid, and later pandemic timepoints [main topics]) and comparing them to transcript content. Summary reports and illustrative quotes were also reviewed with the full RESTORE study team for further input and refinement. As a form of member checking the trustworthiness of our analysis process, these findings were then shared with a subset of interview participants at an Advisory Board meeting, where feedback and confirmation was obtained that findings accurately reflected staff efforts and experiences when trying to execute cancer screening services during distinct phases of the pandemic. The full analytic process took approximately 12 months, resulting in a final content summary document that serves as the basis for this manuscript. Additionally, to support rigor in the presentation of our findings, the Consolidated Criteria for Reporting Qualitative Research (COREQ) was employed as a guide [26].

## Results

We conducted interviews with 20 of the 26 individuals identified; the six who declined cited time constraints. The 20 study participants represented a broad range of clinic and leadership positions and played various roles in CRC screening provision and pandemic-related outreach **(Table 1)**. Thirteen participants were directors, supervisors, or managers, many of whom also served as clinicians or nurses. Seven participants were frontline patient-facing staff such as medical assistants, health educators, referral coordinators, and front office staff. Other participants held roles in CRC screening decision-making and execution and/or clinic management of COVID-19 service provision in at least one of the following areas (note that participants can represent multiple roles): CRC education, referrals, and scheduling (8); patient access and service (4); population health and quality (3); patient safety (3); medical informatics and analytics (3); and operations (2). Overall, participants ranged in age between 26–56, and most were female (85%).

**Table 1:** Description of interview participants.

Descriptors (N = 20)	N (%)
***Positions***	
Director, Manager, Supervisor, Provider*	13 (65%)
Other Frontline, Patient-Facing Staff	7 (35%)
Roles In CRC decision-making/ execution**	
CRC*** screening (*education, referrals, scheduling*)	8 (40%)
Patient access and service	4 (20%)
Population health and quality	3 (15%)
Patient safety	3 (15%)
Medical informatics and analytics	3 (15%)
Operations	2 (10%)
**Sex**	
Female	17 (85%)
Male	3 (15%)
**Age**	
Average	41
Range	26–56

*Can include clinicians and nurses; ** Participants can represent more than one role;

*** CRC = colorectal cancer. ***Note****: race/ethnicity data was not collected for interview participants*

**Fig 2:**
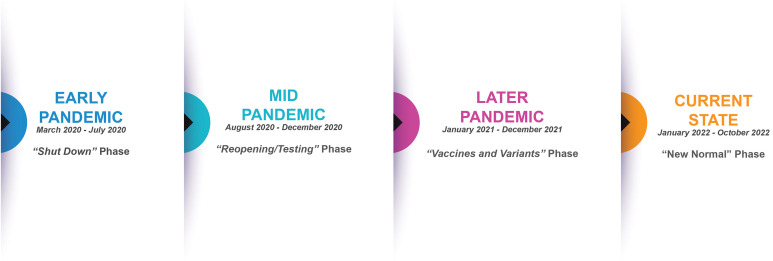
COVID-19 phases.

Below, we describe impacts of the COVID-19 pandemic on the FQHC’s ability to provide CRC screening services and education. Based on our content summary, we organize our findings across three topical areas of service delivery along the screening continuum: 1) Changes to in-clinic-delivered care; 2) Organizational capacity for centralized screening outreach; and 3) External capacity for CRC testing and procedures. The first two topics focus on distinct aspects of CRC screening service delivery in primary care: visit-based test distribution and centralized outreach. The third topic addresses COVID-19 impacts on FIT suppliers and specialty gastroenterology care sites. Within each topic area, we describe the organization’s experiences across three distinct time periods of the pandemic: the “shutdown,” “reopening,” and “vaccine” phases from March 2020 – December 2021. The current state (at the time the interviews were conducted) is also included (Fig 2). Finally, we summarize broader lessons learned and note actions taken to improve CRC screening efforts over the course of the pandemic.

### Changes to in-clinic-delivered care

#### The FQHC was overwhelmed by respiratory infections, and prior disaster plans offered minimal guidance.

During the pandemic, the organization’s ability to provide routine and preventive care was severely limited, requiring a substantial shift toward pandemic management and telehealth services. Prior to the pandemic, the FQHC had well-established yearlong “roadmaps” designed to guide CRC screening efforts and goals. However, during the shutdown and reopening phases of the pandemic, all cancer screening “happened on the side” when feasible according to surges in infection rates, access to patients, screening tests, and overall staffing capacity. One participant reflected, *“At times, when our clinics were being overrun by URI [upper respiratory infection] and COVID, we couldn’t do anything else.”*

Throughout the early and middle timepoints of the pandemic, COVID-related clinic policies such as masking, temperature checks, or waiting for appointments in their cars rather than in the clinic lobby, created patient resistance to obtaining needed care, including CRC screening. A participant described, “*In some cases [patients] weren’t necessarily fearful of coming or leaving the house, but they would approach the clinic and things were different. They’re being scanned outside, and they’re being asked to wait in their cars. Some of our patients didn’t understand that process and it was difficult.”*

#### The FQHC lacked infrastructure for rapid telehealth expansion, and challenges arose with building patient rapport and creating new workflows.

The FQHC launched telehealth during the early phase of the pandemic, constituting a significant shift in care provision. As one participant recalled*, “By April of 2020 we were 80% telehealth and 20% face-to-face… So it was a pretty rapid change, but [the organization] implemented it very quickly.”* Participants described how this shift required a “huge lift” by the organization to develop and train both providers and patients on using telehealth and overcoming technology issues. Patients preferred to “wait” to see a provider in person and lacked the technology needed for video-based visits. The short, phone-based visits hindered interpreter services and tended to exacerbate language barriers. Staff observed that telehealth appointments focused primarily on acute needs and COVID-related symptoms, leaving little time to address cancer screening gaps. Thus, throughout the shutdown and into the reopening phases of the pandemic, providers struggled to create rapport with patients over the phone, especially given the lack of video technology. A participant summarized, “*I think the biggest adjustment for our staff was telehealth. Going from face-to-face visits, having virtual visits - there was a workflow change, there was a system change. Processing and building those relationships with patients over the phone, that was something we were having to adapt to.”*

Demonstrating how to do FIT testing was not possible using phone-based telehealth, as a participant noted, *“The clinics have always said having the patient in-person and being able to demonstrate how you brush the stool, what you do after, how you put it on the card, is central to having lower error rates. And so obviously that’s gone in telehealth.”* Additionally, given limited in-clinic visits, staff could only order FITs as patients became due, requiring mailing kits and making reminder calls. These follow-up efforts rarely occurred due to staffing constraints and COVID-related priorities. As one participant described, *“What we found out after the fact was that a lot of orders were being placed for FIT kits for patients, but nobody was sending the FIT - so that was a gap in our telehealth process.”*

#### Telehealth improvements eventually expanded patient outreach.

Throughout the latter half of the reopening phase, telehealth services improved significantly and enabled reach to patients who had experienced barriers to access before the pandemic. A participant noted, *“[The health center] primarily services low-income areas with high health disparities so getting patients to come in for anything has always been traditionally very hard for us. That’s why telehealth was a great option for those patients.”* Furthermore, patients gradually became more comfortable with telehealth. A participant observed, *“A lot more patients were preferring telephone visits. Especially the ones that had a fecal test came back abnormal and they needed an appointment to have a referral placed. Instead of them physically coming into the office, staff would just call them over the phone… A lot of the patients were grateful for that.”*

Moreover, as staff gained experience with telehealth over time, centralized teams were able to support the mailing of FITs based on orders placed during telehealth visits. Additionally, some clinical teams introduced innovative strategies to encourage patient participation, including gift-card incentives. As one participant described, *“We would mention if they would bring back the [FIT] envelope with the [stool] sample that we would give them a gift card…they didn’t even have to come in-person, they were able to go through the drive thru and hand it to us. A lot of patients, when they heard they would get something in return, would do that.”*

Over time and as in-person visits became more accessible in the later phase of the pandemic, tensions emerged between providers and leadership about telehealth usage. A participant described, *“There was a sense that telehealth was here to stay…[but it] felt like we were kind of overusing it. We were up to 40% at one point [in 2021]. It shouldn’t replace in-person obviously, [there is] a sub-set of patients whose care will never be able to get fulfilled via telehealth, like diabetics.”* By mid-2022, the FQHC had stabilized, with telehealth accounting for about 20% of visits and clinical teams returning to distributing FIT kits during in-person care.

### Organizational capacity for centralized screening outreach

#### Low staffing limited centralized outreach.

In the shutdown phase of the pandemic, as staff transitioned to remote work options to comply with stay-at-home policies, clinics had limited capacity to focus on CRC screening. As one participant observed, *“Everything just went on the back burner - we had less staff, less providers at the clinic, and most of our focus was on urgent episodic visits versus any preventive or maintenance [care].”* Thus, centralized quality staff who managed CRC screening efforts, particularly the mailed FIT outreach program, were reassigned to support the overwhelmed call center handling COVID-related inquiries. This resulted in a pause in CRC outreach campaigns such as mass FIT mailings, outreach calls, educational tabling events within clinics, and broader community events to promote CRC screening and encourage FIT completion. A participant described, *“This department would hold a lot of [CRC] outreach attempts and run a very fluid [CRC] outreach program - and when COVID started that was a very big part of this department that was impacted. We weren’t sending out educational fliers to patients anymore, because of the low bandwidth and a lot of limited access within the team. We weren’t even able to do [CRC] outreach attempts with patients, like, calling.”* As a result, providers and their teams became primary sources of CRC screening education and promotion. As the pandemic wore on, these challenges were compounded by the fact that the FQHC was now serving more patients, because of job loss and the loss of employer-provided insurance.

#### New barriers to mailed FIT outreach arose.

Although the organization resumed its annual mailed FIT outreach program in summer 2020 during the reopening phase, and mailed out approximately 13,000 FITs, significant barriers to screening persisted. Participants observed that many patients were no longer living at their home address and thus did not receive the mailed FIT. A participant described, “*We mailed it [FIT] to their home. Because of the lockdown [patients] weren’t living at their homes, or they hadn’t been at their home for weeks. So there were…a lot of people moving around, a lot of people not wanting to go out to their mailbox.*” In addition to COVID-related fears about leaving their houses, staff reported that masking and social distancing policies impeded patient drop-off of completed FITs at their clinic.

#### EHR dashboard and workflows for mailing FIT from telehealth visits improved outreach.

During the later pandemic phase*,* centralized outreach teams gradually came back together. The quality team developed dashboards in the new EHR system (Epic was installed in August 2019) to assist provider teams in tracking patients due for CRC screening. Providers’ orders for FIT placed during telehealth visits were now supported by the centralized registry team, which mailed FITs to patients’ homes*.* One participant noted*, “We told our providers again when they were on a telehealth visit to go ahead and place the order for FIT kits during the visit. And the registry team would then mail it out to the patient so patients would get it at home.”*

By the vaccine phase of 2021, the mailed FIT outreach program had been fully re-activated and now included an “opt-in” text message prior to mailing the FIT to increase engagement. As a participant described, “*We’re about back up to speed pre-pandemic. We resumed our outreach services, our quality team focused on these [cancer screening] measures – sent out text messages, automated calls, ramped up our outreach, and communicated with patients to notify them of the need of their screenings.*” Finally, some improvements were observed in how registry teams outreached to patients, reducing the time to process referrals to GI specialists. A participant observed, “*It was much better. The patients were coming, we were calling. We would get the FIT kits, get the list of who was missing their cancer screening. There was now somebody dedicated to doing outreach three days a week.”*

#### Staffing challenges hampered CRC screening education, outreach, and follow-up.

During the shutdown and reopening phases, centralized outreach efforts were severely constrained by ongoing staffing shortages and COVID testing demands, limiting CRC educational and awareness campaigns. The staffing challenges, along with limited clinic hours, also created backlogs in departments such as those managing referrals, including delays in processing referrals for follow-up colonoscopies from abnormal FIT results. By the first half of 2021, a significant backlog emerged, as described: *“We saw a lot of patients who had an abnormal [FIT] who never followed up. For the outreach team in April of 2021, there was a backlog of 300 to 400 patients who hadn’t taken action on their abnormal tests.”* Throughout the ebbs and flows of the pandemic, managing variants and new surges, the impact on staffing was profound. Staff turnover reached unprecedented levels, with one participant describing the situation: *“We also had furloughs of staff. Some staff left on their own or they were let go because we had to relook at finances so that we could continue to keep the operations going - we were definitely left with less staff in the end.”* Throughout the later phase of the pandemic, staffing was described as the “worst ever seen” due to burnout, illness, resignations, and redeployments to vaccine centers. This was an issue for provider teams and also for patient education and community awareness campaigns. It was noted by participants that both staff and patients were in need of re-education and re-training on cancer screening importance and guidelines.

However, by later 2021, the centralized team was fully staffed and could begin to address the referral backlog and new referrals. A participant noted the improved registry team capabilities: *“The registry [team] was in place, and we were able to track [patients]... call them the first time, give them their results, tell them about their referral. If they needed anything, if there were any barriers we were still able to help them. If they don’t have transportation, we can provide them with transportation.”* Additionally, during this time, patients began to slowly return to routine care, providers offered screening during in-person visits, and patients were more willing to complete and return FITs. Text-based reminders were now commonly sent to patients who were offered a test. A participant summarized this late pandemic stage: “*I think in 2021 we had a lot of catch up to do…we were doing huge outreach, number one for the vaccines. Number two, the vaccine was also an opportunity to reengage patients.*” As new staff were hired or promoted during 2021 and into 2022, the FQHC sought to cross-train centralized staff in multiple outreach topics, allowing for bundled outreach and protections against staffing reductions.

### External capacity for CRC testing and procedures

#### Limited availability of stool test kits and reduced gastroenterology capacity thwarted screening delivery.

The provision of CRC screening services during the pandemic was heavily impacted by both supply-chain issues with FITs and limited accessibility to externally contracted GI specialists who provided colonoscopies for FQHC patients. Early in the shutdown and reopening phases of the pandemic, the vendor that supplied FITs to the organization pivoted to producing and distributing COVID-19 test kits. As one participant explained, “*Often times the supply chain got broken. [The vendor] where we got our FITs was so inundated with COVID swabs and COVID supplies, there was a while we were limited on how many FITs they would give us.*” These issues subsided during the vaccine phase of the pandemic.

Referring and scheduling FQHC patients for follow-up colonoscopies after abnormal FIT results was challenging throughout the pandemic, as external GI specialists had limited capacity. In the early shutdown and reopening time points, participants reported experiencing limited response from GI facilities when reaching out for a colonoscopy referral, describing, *“It really depended on the [GI] facility, many would take days and weeks [to respond to referrals], and in some cases [GI] specialists were not available at all.”* This was further exacerbated by limited staffing at insurance companies, which led to longer-than-usual delays in obtaining service authorizations. Many colonoscopy appointments scheduled prior to the shutdown phase were cancelled or “became lost” with no follow-up from the GI office for several months to a year. A participant observed, *“Patients who had their colonoscopies scheduled for some time [prior to pandemic], they got canceled. And a lot of those patients lost track of going back, calling their doctors back and saying, ‘It’s been a whole year, can I get my colonoscopy now?’ It was like it [colonoscopy] was forgotten.”* Issues accessing colonoscopy sometimes led providers to offer a repeat FIT as an interim solution. As a participant described, “*I actually had a couple [patients] with positive FITs, and it was again a risk-benefit discussion with them of what is their comfort level, which way to go…And in a few cases ended up repeating the FIT knowing it’s not the best but it provided some potential reassurance.*”

Furthermore, during this time, participants observed patient reluctance to go to GI offices for fear of COVID exposure for themselves or their caretaker. A participant described, “*Patients with abnormal FIT just prior to pandemic awaiting colonoscopy or screened during pandemic with FIT were deferring colonoscopy if available due to COVID fears and desire to wait for improvements in the pandemic.”* Many GI offices at this time were not offering telehealth for pre-consultation visits. A participant noted this challenge, stating, “*That’s one of the barriers I have encountered with GI specialists that they are not doing it [telehealth]. I haven’t really seen it happening very much. It’s almost like they want to see patients in person, and they don’t offer telephone appointments.”* These disruptions continued during the reopening phase, where GI clinics continued to be either closed, have limited hours, see only emergency cases, or have appointment backlogs of over six months. A participant observed, “*We were still seeing the long delays with [GI] specialists. There were so many that were completely closed down at that time. And the ones that stayed open, the wait [time], it was months to see a specialist at that point.”*

Through the first half of 2021 and the vaccination phase of the pandemic, GI offices had specific and often varying COVID testing and vaccination policies which could hinder patient follow-through. A participant described, “*I think by this time, it was a little more difficult because patients had to prove things like a vaccine or negative [COVID] test so many days before the colonoscopy procedure*.” Participants also noted that women at times were unable to attend on-site care appointments, such as with GI specialists, due to providing at-home care for children attending online school. By later 2021, participants described minor improvements in access to follow-up colonoscopies as more GI offices re-opened or improved staffing. A participant that assisted with managing abnormal FIT results described, “*[GI] offices were also opening up more, so they were able to schedule with the specialist. In 2021 it was a little bit easier for them to actually do the consult. I think it [wait-times] went from six months to three months.”* While access eased somewhat, in 2022 participants reported that wait times for some contracted GI practices were still often up to four months.

#### Community resources to support colonoscopy receipt were scarce but eased over time.

During the initial shutdown phase and into early 2021, participants observed a reduction in community or clinic-based resources (e.g., help with cost or transportation/ companion needs) to assist the patient with obtaining a follow-up colonoscopy. A participant commented*, “We would let patients know they should call [GI office] and ask the [colonoscopy] prices and see if it was something they could afford… A lot of the time patients didn’t follow through; it was too expensive and time consuming.”* However, by mid-2021 and into 2022, participants started to experience improvements in finding patient resources to facilitate colonoscopy completion, such as resumed transportation options or locating programs to aid in defraying the cost of the procedure. Additionally, the FQHC received a grant during this time to support hiring a nurse who would contact patients with an abnormal FIT result and offer navigation services. This included support with scheduling, bowel preparation, and overcoming logistical barriers such as transportation, to ensure patients followed through with colonoscopies.

#### Changes to CRC screening recommendations led to overwhelm.

During the vaccine phase of 2021, the number of eligible patients for CRC screening increased after guidelines for starting CRC screening were lowered to age 45. A participant described the ongoing impact of this shift as such: “*We had a challenge thrown at us with colorectal cancer screening, now it’s opened up to 45-49, so we just added 15,000 people to our denominator. And our data shows that typically the 50–60-year-olds are the least compliant… we already know the younger population doesn’t do this, [so] how do we get the even younger population to do it?”* Due to this change in CRC screening guidelines, along with the ongoing pandemic impacts on staffing and capacity, participants expressed worry that the FQHC might never be able to become “caught up” on CRC screening for their patient population.

### Actions and suggestions for improvement

**Fig 3:**
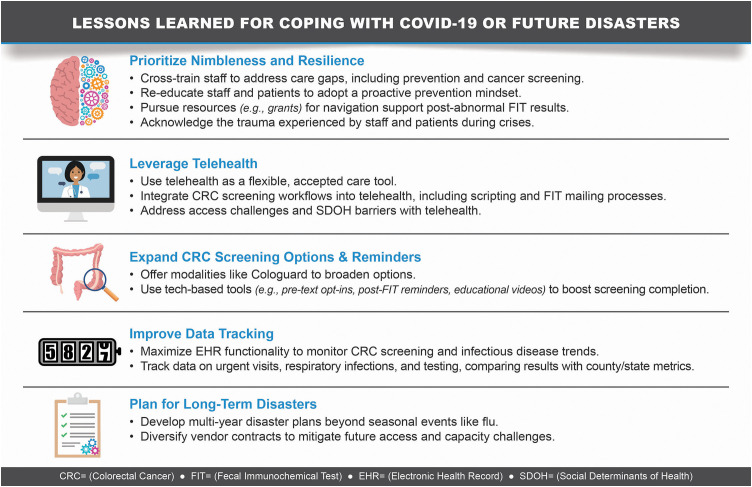
Lessons learned for coping with COVID-19 or future disasters.

Participants shared their observations of various actions taken or planned to counter the two-year impact of the pandemic on the FQHC’s CRC screening efforts. These strategies and potential improvements are described below and highlighted in Fig 3.

#### FQHC expanded stool-test options.

Concerns continued to grow that the FQHC might never return to pre-pandemic CRC screening levels given the compounding nature of care delays, lowered CRC screening age, and increases in the FQHC’s patient membership during the pandemic. Hence, one method the FQHC employed in mid-2022 to increase CRC screening rates was to introduce another at-home screening method besides just FIT. The FQHC implemented an additional stool-based FIT-DNA test (which detects both blood and DNA markers in stool) that has a longer, every-three-year, screening interval. An operational leader described current and future efforts: “*I think having Cologuard as an option for us has been huge because at least it’s good for 3 years. It’s an easier sell to the patients too.”*

#### FQHC deployed automated patient outreach tools and re-trained staff.

In the latter phase of the pandemic, the organization continued to develop and test new CRC screening approaches to manage the CRC screening backlog and facilitate test completion. These included ongoing use and refinement of the “opt-in” text approach as part of their broader mailed FIT campaign and improving colonoscopy referral. A participant noted, “*This year I’ve been doing a monthly opt-in. So, we do text messages to about 2000 patients a month. If they opt-in to receive a FIT kit, we send them that FIT kit and then we do a follow-up additionally. That’s increased our compliance rate.”* Furthermore, both new and prior staff were re-trained regarding the importance of CRC screening and related workflows, and broader health education campaigns were planned to motivate patients to focus on preventive care. One participant described these efforts as thus, “*We have spent an incredible amount of time working on making sure our Epic [electronic] care gaps are firing appropriately, and [making it] easy to see and link to the last test quickly for our providers. And also that our physicians are aware of what are the clinical guidelines triggering those care gaps for the cancer screenings. We have focused a ton of time on training and monitoring on where to go to look at what patients are due [for CRC screening].”*

#### FQHC improved monitoring of infectious diseases and staffing to guide strategic capacity decisions.

Toward the end of the pandemic and into 2022, the organization sought to improve data-tracking tools for short-and long-term planning for infectious diseases including COVID-19, the flu, and emergent infections such as respiratory syncytial virus (RSV). Data points, staffing, and capacity issues were now assessed on a regular basis rather than during seasonal time periods. A participant described these ongoing efforts this way: “*Now we learned a lot. We’re in a very different place than we were in the beginning… We basically have systems in place where we have thresholds for what does it look like, what are the data points that we’re always monitoring…I think the difference is that [prior] winter [surge] planning used to just be winter, now we’re doing this every day, every week, where we’re looking at how many patients are calling in reporting URI symptoms. How many walk-in and urgent care visits are we seeing? How many COVID tests are we giving? And comparing that to county data, [and] we’re doing that on a weekly basis. And then what are all the action steps that we can take if we have more demand than supply…And we are looking at that now with mpox and also flu.”*

#### FQHC aimed to broaden vendor and specialty care partnerships.

The organization was also re-evaluating their disaster planning approach to now include long-term disaster planning rather than the prior efforts that focused on short-term events like the flu season. This effort included expanding and diversifying partnerships and contracts with specialty vendors, such as GI clinics, to lessen extended access and capacity issues during future disasters. The FQHC was also considering the option of bringing a GI specialist in-house to increase capacity, as noted by one participant: “*I think we definitely need to be able to expand our [specialty] services more. I know the FQHC is also working to get a GI specialist in one of the clinics, so that would help with the abnormal FIT [results].”*

#### Importance of being nimble and resilient.

Finally, despite the many challenges experienced over the two years, participants described how the FQHC learned to be both flexible and resolute in its ongoing efforts to provide care and CRC screening services throughout various phases of the pandemic. Participants observed how the organization gained experience in managing the ebbs and flows of the pandemic, learning to adjust its care approach according to its disaster plan and current conditions. Telehealth became a permanent and essential care tool that was accepted by both staff and patients and could be activated or scaled as needed according to the impacts of the pandemic or other emergent issues on access and capacity. Overall, the organization’s ability to adapt during various phases of the pandemic was summarized as follows, *“We’ve also learned about the resilience we have to deal with so much and keep on going…. I feel it’s actually put more focus on what we need to do and how we need to reach our patients in different ways and not just one size fits all. So I am not saying it’s good to have a pandemic, it was terrible what everybody went through, but I think we’ve come out on the positive side of it in the end.”*

## Discussion

COVID-related challenges to delivering CRC screening and follow-up experienced by a large, urban FQHC included being overloaded with upper respiratory infections, lacking infrastructure to immediately expand use of telehealth, and having little guidance from previously existing disaster plans that focused on short-term, episodic issues (e.g., flu season). Ongoing staffing challenges and patient hesitancy to attend clinic visits were also barriers. Limited availability of stool test kits and reduced gastroenterology capacity further hampered CRC screening delivery. Based on our interviews with FQHC staff, several elements of clinic operation emerged as important steps to ameliorating these challenges: continued and flexible use of at-home stool-based testing options, maintaining infrastructure for ongoing telehealth use, utilizing automated patient-outreach tools, improving tracking systems to support strategic staff deployments, and building partnerships to ensure access to specialty care services. Our findings can guide health systems serving vulnerable populations in preparing for future care disruptions, such as pandemics or natural disasters.

Findings from prior studies and the experiences of our partnering FQHC underscore the importance of home-based cancer screening options in maintaining screening participation during health care disruptions [27,28]. FIT testing was promoted by national organizations for first-line screening in the early months of the pandemic because tests could be mailed to patients and mailed back to the clinic, obviating the need for in-person visits unless test results were abnormal [29]. Fisher-Borne and colleagues used data from a national collaborative of FQHCs and reported that, while 50% of interviewed FQHCs stopped offering CRC screening, greater proportions – 77% and 90% – stopped offering breast and cervical cancer screening, respectively, suggesting that the at-home stool test option for CRC screening mitigated COVID-related declines [30]. Our findings that stool-based approaches were thwarted by supply chain issues, patients not being at their home address, and the need to develop workflows to mail tests ordered during telehealth visits, suggest that additional efforts may be needed to maintain the effectiveness of these programs during pandemics and other natural disasters. Moreover, further investments in home-based self-sampling technologies should be made (e.g., self-collection of HPV DNA testing); these investments can take the form of funding for research to support test development and approval, test promotion, and patient education and access.

While participants initially found the telehealth transition challenging, they recognized its significant potential benefits. According to the National Association of Community Health Centers, 95% of FQHCs utilized some form of telehealth during the first year of the pandemic [31]. This highlights the importance of maintaining flexible health care infrastructure that can be quickly adapted to emergency conditions [32]. Developing robust telehealth capabilities emerged as a critical component of long-term disaster preparedness, particularly when in-person clinic visits become difficult, potentially high-risk, or impractical. Finally, the pandemic prompted the introduction of new health center tracking systems that monitor and report reasons for urgent care visits and calls to the call center, COVID-19 testing and positivity rates, and other factors. Clinic staff expressed a strong interest in standardizing and expanding these tracking systems to include emergent infections. These new tracking capabilities could enable more strategic staff deployments, including cross-training initiatives that would allow for rapid reallocation of personnel during future health care emergencies. By understanding patient care patterns in real-time, FQHCs could become more agile and responsive to emerging health challenges.

Another important change is that, as centralized cancer screening outreach teams re-formed, they relied more heavily on automated approaches (i.e., text messaging, automated phone calls) to remind patients to complete CRC screening. Prior literature has highlighted the benefits of using automated or digital approaches for reminders and patient education in place of live outreach calls to reduce demand on clinic personnel who may be needed for other urgent care roles. Instructional videos sent using automated text messages or patient portals may reduce the need for live phone call outreach [33–35]. Virtual navigation approaches are also being tested that provide education and reminders to patients who need to undergo a colonoscopy procedure [36,37]. Integrated models that offer bundled services (offering CRC screening or reminders during other preventive care visits) are also emerging as a cost-effective approach that can minimize health system and patient burden while addressing health disparities [38]. One bundled strategy implemented by this FQHC, due to their experience with the pandemic, involved cross-training centralized staff to address care gaps across multiple preventive health needs (i.e., cancer screening, vaccinations) in a single phone call.

The colonoscopy backlog presented important challenges to accomplishing screening and follow-up for individuals with abnormal test results, an issue that was further exacerbated by USPSTF guidelines that dropped the CRC screening initiation age to 45 (resulting in more than 18 million people nationwide becoming newly eligible for screening) [39]. Other actions could help ease the ongoing backlog of GI services [9]. These include expanding use of telehealth for pre-procedure visits, expanding health care access via expanded clinic or GI hours, or identifying donated GI services for patients who receive care in community clinics. GI policies to prioritize high-risk and FIT+ colonoscopies could reduce scheduling wait-times for these higher-risk patients [40]. This is important because 7–12-month screening delays will elevate the proportion of CRCs detected in more advanced stages (from 26% to 29%), which worsens to 33% after a 12-month delay [14]. Additionally, creating back-up plans for staffing positions vacated during the pandemic is also needed. Finally, FQHC staff desired more and greater diversity in the number of external GI contracts to better meet the needs of patients. Future research should explore how to coordinate disaster preparedness plans across primary and specialty care sites to ensure a synergized focus on high-value care delivery.

### Limitations

Our study has several limitations. We may not have interviewed all relevant staff with pertinent knowledge of CRC screening efforts during the pandemic, and high staff turnover introduced additional recall challenges. However, our sample size was sufficient for obtaining consistency in responses and breadth and depth of experiences [19]. Additionally, we engaged in a member-checking process by sharing back a summary of findings with a sub-set of interviewed participants to check our interpretations and adjust any missing or incorrect descriptions [41,42]. This, along with our trained interview staff, consistent use of an interview guide, and robust analytic approach, allowed for rigor and consistency in findings. Finally, our study was conducted in a large urban FQHC, and findings may not be applicable to other settings or FQHCs, particularly rural ones. Indeed, the 2-year, 20-percentage point drop in CRC screening in our partnering FQHC (63% in 2019 vs. 42% in 2020) followed by a steady rebound (recovering to 51% in 2021) was more pronounced than the 6 percentage point drop among FQHCs nationally (46% in 2019 vs. 40% in 2020 vs. 42% in 2021), suggesting greater impacts and a stronger rebound in our partnering FQHC than at most other FQHCs [15].

## Conclusion

Despite the many challenges experienced by clinic staff during the pandemic, interviewees described how the FQHC successfully adapted to continue providing care and CRC screening services throughout the pandemic phases. Challenges faced in both internal and external settings could be addressed through continued and flexible use of at-home stool-based testing options, maintaining infrastructure for continued telehealth services, utilizing automated patient outreach tools, improving tracking systems that can support strategic staff deployments, and bolstering partnerships to ensure access to specialty care. Disaster preparedness plans should prioritize these approaches to maintain CRC screening participation during natural disasters.

## Supporting information

S1 Appendix Interview guide sample. (DOCX)

S2 Appendix Extraction template for summarizing interviews. (DOCX)

S3 Appendix Minimal anonymized qualitative data set. (PDF)
