# Supplementary material for: Impact of COVID-19 on colorectal cancer screening in a federally qualified health center: Provider and staff perspectives
Source: PLoS One. 2026 Jan 13;21(1):e0340184. doi: 10.1371/journal.pone.0340184 (PMC12798968; doi:10.1371/journal.pone.0340184)
Supplement: S1 Appendix — (DOCX) [file pone.0340184.s001.docx]

**S1 Appendix: Interview Guide Sample**

| Domain | Questions | Probes |
| --- | --- | --- |
| Background | 1. Please describe your current role/position and how long you’ve been with [organization]? |  |
| History of role in cancer screening | 1. What role do you currently play in supporting cancer screening? | 1. Has your role changed over time since you’ve been with the organization? Please describe. 2. Do you play a role in all cancer screening or a specific role in particular cancer screening? |
| Pre-COVID (prior to March 2020) | | |
| Health system barriers | 1. What did you observe or experience as common health system challenges at your FQHC* to cancer screening prior to COVID 19? 2. What was typically working well to facilitate cancer screening prior to COVID 19? | 1. Were there challenges pertaining to education of patients regarding importance of cancer screening; workflows to order/schedule/complete screenings; actual completion of screening and follow up of results (normal and abnormal), staffing, community resources? 2. Ask for both cancer screening in general and for CRC**. 3. How, if at all, were those challenges typically addressed? |
| Patient barriers | 1. What did you observe or experience as common patient challenges to cancer screening prior to COVID 19? 2. What was typically working well to facilitate cancer screening prior to COVID 19? | 1. What was hard/missing for cancer screening steps: education; scheduling; completing; communicating results (normal or abnormal): referrals to specialty care for abnormal result. 2. Ask for both cancer screening in general and for CRC. 3. How, if at all, were those challenges typically addressed? |
| Questions in this section were asked for 3 phases of the pandemic: Early (March - July 2020), Mid (August - December 2020), Later COVID (2021) | | |
| Service impact | 1. During the [early, mid, later] phase of the pandemic, what occurred with cancer screening services? | 1. Did they continue in a modified version or stopped all together? 2. Ask for both cancer screening in general and for CRC. 3. Did they remain similar to the previous time period, or did things improve or get worse? 4. Ask about impacts for cancer screening steps: education; scheduling; completing; communicating results (normal or abnormal): referrals to specialty care for abnormal result. |
| Health system barriers | 1. What health system/clinic level barriers or challenges were present regarding cancer screening during this time? 2. What challenges emerged during this time regarding community resources to support your patients in cancer screening services? 3. What challenges emerged during this time for patients with abnormal cancer screening results and the need for a follow-up/referral to a specialty? | 1. Ask about staffing issues (such as work from home, limited staffing due to fear or social distancing, redeployment); lack of PPE; lack of lab services for processing; inability to process referrals; specialty shutdowns, inability to mail out FITs***) 2. Ask about lack of community resources to support screening (transportation, interpreters, financial aid, any issues with accessing or using these services etc.). 3. Were any cancer screening services, pertaining to CRC, harder or easier to continue during the various phases? Please describe. 4. How, if at all, were those challenges typically addressed? |
| Patient barriers | 1. What patient-level challenges were present during this time? | 1. Were any cancer screening services, pertaining to CRC, harder or easier to continue during the height of the pandemic? Please describe. 2. How were these challenges addressed? |
| Protocols/Processes | 1. Were there any protocols or processes put in place during this time to help with or facilitate cancer screening? 2. What role, if any, did a disaster preparedness plan play in identifying and implementing health system level changes to address cancer care during this phase of the pandemic? 3. What was the process for monitoring and tracking health system operations, such as changes to staffing and/or clinic hours, during this phase of the pandemic? | 1. Ask about telehealth, calls, reminders to patients, training other staff, identifying community resources/supports, etc. 2. How were these protocols or processes communicated to you? To external partners? 3. What were staffs’ experiences in implementing these process or protocol changes? Were some harder/easier than others? How successful or not were they? 4. Ask about changes in clinic hours, staff being redeployed from typical role, working from home versus in-clinic, staff quitting or not returning. |
| Telehealth | 1. Please describe in more detail how telehealth was implemented during the height of the pandemic. 2. What changes occurred with telehealth during this phase of the pandemic? | 1. What role did telehealth serve in helping with cancer screening? 2. What successes were experienced with telehealth and cancer screening during this time? 3. What challenges were experienced with telehealth and cancer screening during this time? 4. Ask about changes to processes or protocols with telehealth for mid and later phases: patient/staff reaction (patient barriers, limitation to internet access, tech knowledge, etc.) to telehealth, if use was increasing, decreasing, or staying the same. 5. Particularly for CRC screening and mailed FIT, were any new or different processes put in place to facilitate CRC screening via telehealth? 6. Were any differences observed in patients’ willingness to complete cancer screening, including mailed FITs, using telehealth (pandemic) versus in-person (pre-pandemic)? |
| Guidelines | 1. How did the broader context or environment, such as local and state guidelines regarding COVID-19, influence your FQHC’s approach to cancer screening during this phase of the pandemic? | 1. Ask about elective surgeries and non-essential procedures should be delayed, ACS recommended delaying cancer screenings; California stay at home order March-April, focus on telehealth. 2. To what degree did the broader local and state environment create challenges to cancer screening? 3. How did your FQHC address these challenges? 4. To what degree did the broader local and state environment facilitate ways to implement cancer screening? |
| Current State (Summer / Fall 2022) | | |
| Service impact | 1. How would you describe and assess cancer screening services right now at this point in time? For CRC? 2. What long-term impacts has the COVID-19 pandemic had on your FQHC’S ability to outreach to and/or engage patients in cancer screening, particularly for CRC? *Such as housing and food insecurity; job loss; health care coverage/insurance loss; ongoing inequality in terms of health care/access, etc.* | 1. What is working well and not working well and why do you think that is (Covid or other reasons)? 2. What concerns do you continue to have regarding cancer screening at your FQHC? 3. Would you say cancer screening services “have returned to pre-pandemic times”? Why or why not? |
| Advice/Future | 1. What do you believe is needed now to improve cancer screening services for CRC at your FQHC? For CRC? 2. Is there anything else you’d like to share about COVID-19 impacts on your FQHC’s ability to offer and support cancer screening services to their patient population? | 1. Are these system level needs/changes and/or patient level needs/changes? 2. Are these changes different depending on insurance coverage, language preferences, race/ethnicity? 3. What might facilitate or get in the way of the ideas you are suggesting? |
| Interview recruitment | 1. Is there anyone else we should speak with at your FQHC about this topic? | If yes, what is their name and title/role? |

*FQHC=Federally Qualitified Health Center; ** CRC=Colorectal cancer;* ** FIT=Fecal immunochemical test
