# Supplementary material for: Impact of COVID-19 on colorectal cancer screening in a federally qualified health center: Provider and staff perspectives
Source: PLoS One. 2026 Jan 13;21(1):e0340184. doi: 10.1371/journal.pone.0340184 (PMC12798968; doi:10.1371/journal.pone.0340184)
Supplement: S2 Appendix — (DOCX) [file pone.0340184.s002.docx]

**S2 Appendix: Extraction template for summarizing interviews**

| **Topical areas and sub-categories** | **Extracted data from transcript (quote) with participant ID** | **Interviewer notes (if needed)** |
| --- | --- | --- |
| **Background/role**  -Formal position  -CRC* screening role  -Other roles  -Changes in roles over time | “xxxxx” – ID 101  “xxxxx” – ID 102  Etc. | (e.g. ppt 101 had major role in CRC screening during early pandemic) |
| **Pre-COVID pandemic time period (prior to March 2020)** | | |
| **CRC screening barriers**  -Health center level  -Patient level  -Specialty/Vendor level (e.g. *GI**offices, FIT*** vendors*)  -Other or additional information | “xxxxx” – ID 101  “xxxxx” – ID 102  Etc. |  |
| **CRC screening facilitators**  -Health center level  -Patient level  -Specialty/Vendor level (e.g. *GI offices, FIT vendors*)  -Other or additional information | “xxxxx” – ID 101  “xxxxx” – ID 102  Etc. |  |
| **Early COVID/ shut down phase (March-July 2020)** | | |
| **CRC screening barriers**  -Health center level  -Patient level  -Specialty/Vendor level (e.g. *GI offices, FIT vendors*)  -Other or additional information | “xxxxx” – ID 101  “xxxxx” – ID 102  Etc. |  |
| **CRC screening facilitators**  -Health center level  -Patient level  -Specialty/Vendor level (e.g. *GI offices, FIT vendors*)  -Other or additional information | “xxxxx” – ID 101  “xxxxx” – ID 102  Etc. |  |
| **Telehealth**  -Description/history  -Barriers  -Facilitators | “xxxxx” – ID 101  “xxxxx” – ID 102  Etc. |  |
| **Other impacts on care at FQHC******  -Health system operations  -Communications regarding protocol changes  -Changes to clinic staffing, redeployment, PPE, work from home  -Other | “xxxxx” – ID 101  “xxxxx” – ID 102  Etc. |  |
| **Mid-COVID/ Re-opening and testing (Aug-Dec 2020)** | | |
| **CRC screening barriers**  -Health center level  -Patient level  -Specialty/Vendor level (e.g. *GI offices, FIT vendors*)  -Other or additional information | “xxxxx” – ID 101  “xxxxx” – ID 102  Etc. |  |
| **CRC screening facilitators**  -Health center level  -Patient level  -Specialty/Vendor level (e.g. *GI offices, FIT vendors*)  -Other or additional information | “xxxxx” – ID 101  “xxxxx” – ID 102  Etc. |  |
| **Telehealth**  -Evolution/ changes over time  -Barriers  -Facilitators | “xxxxx” – ID 101  “xxxxx” – ID 102  Etc. |  |
| **Other impacts on care at FQHC**  -Health system operations  -Communications regarding protocol changes  -Changes to clinic staffing, redeployment, PPE, work from home  -Other | “xxxxx” – ID 101  “xxxxx” – ID 102  Etc. |  |
| **Later COVID/ Vaccine and Variants (Year of 2021)** | | |
| **CRC screening barriers**  -Health center level  -Patient level  -Specialty/Vendor level (e.g. *GI offices, FIT vendors*)  -Other or additional information | “xxxxx” – ID 101  “xxxxx” – ID 102  Etc. |  |
| **CRC screening facilitators**  -Health center level  -Patient level  -Specialty/Vendor level (e.g. *GI offices, FIT vendors*)  -Other or additional information | “xxxxx” – ID 101  “xxxxx” – ID 102  Etc. |  |
| **Telehealth**  -Evolution/ changes over time  -Barriers  -Facilitators | “xxxxx” – ID 101  “xxxxx” – ID 102  Etc. |  |
| **Other impacts on care at FQHC**  -Health system operations  -Communications regarding protocol changes  -Changes to clinic staffing, redeployment, PPE, work from home  -Other | “xxxxx” – ID 101  “xxxxx” – ID 102  Etc. |  |
| **Current state at time of interview (summer/fall 2022)** | | |
| **Current state**  -Description  -Ongoing barriers to CRC screening  -Reflections on long-term impacts of pandemic on CRC screening  -Improvements/future plans | “xxxxx” – ID 101  “xxxxx” – ID 102  Etc. |  |
| **Impact/ Influence of external factors**  -Internal disaster plan  -Federal, state, and local policies or recommendations  -Recommendation of other national health organizations (e.g. ACS) | “xxxxx” – ID 101  “xxxxx” – ID 102  Etc. |  |

* CRC=Colorectal cancer; ** GI=Gastroenterologist; *** FIT=Fecal Immunochemical test; ****FQHC=Federally Qualitified Health Center
