# Supplementary material for: Impact of COVID-19 on colorectal cancer screening in a federally qualified health center: Provider and staff perspectives
Source: PLoS One. 2026 Jan 13;21(1):e0340184. doi: 10.1371/journal.pone.0340184 (PMC12798968; doi:10.1371/journal.pone.0340184)
Supplement: S3 Appendix — (PDF) [file pone.0340184.s003.pdf]

S3 Appendix: Minimal anonymized qualitative data set

Pre-Covid Phase

CRC cancer screening barriers

FQHC:

- But with clinics I think the issue is as a clinic it was difficult to resource these initiatives. They tend to be pretty sparse in resources, so for example the person that might have been doing this initiative is an MA but then two people call out right, so then that person gets pulled. 101
- So one of them that is part of the PROMPT study was centralized mailing done once a year. Mass mailing to thousands of patients, consistent calls done after a text message reminder, and a text message prompt primer. So we... that has continued even post PROMPT. So, what we see now is it's a pretty significant number as far as the number of FITs we get back in a year, as a good proportion they represent a good amount 20-25%. They're expensive of course and so you also think we got 30% of them back, oh we sent 12,000 so that means thousands upon thousands are still out there. They were expensive to send out and we didn't get them back. 101
- So that's sort of the FIT component. But we also as a screening option should be offering colonoscopy. The issue is that with giving out a FIT kit clinics and provider teams are allowed to be payer agnostic, so if a patient doesn't have insurance we pay for the cost of the FIT kit, so providers and clinic teams don't have to look whether to see if a patient insured to be able to give to them. And they do have to do that colonoscopy because very few patients are willing to pay cash for colonoscopy. So as a result, some patients... the way the workflow works is the patient comes into the clinic, they speak to the back office or LVN who is rooming them. The LVN is supposed to close as many care gaps as possible. And part of that includes offering them a FIT test or colonoscopy. If they agree to a FIT test, then they get one then and there. They're supposed to get educated on the process and if they don't want to do a FIT test then they talk to the provider about doing a colonoscopy. So it's kind of an opt in method for colonoscopy. 101
- So, there's so much attention that's been thrown on that, so it is frustrating that we haven't see improvement. We've seen little success sending them a FIT kit and then calling them to remind them they have it and offering any help with any [Vendor]ions has been successful at time. 102
- You realize you have to meet people where they are. From an organizational perspective I don't know that we ever really met people where they are. 104
- The video is located on the [Vendor] website and then they were able to save it on the desktop. So, we would just open it directly and it would show the patients the video in English or Spanish and it would explain to them a little bit why it's needed and how it's done. And then I guess they updated the computers at the clinics, and it wiped everything away. Now I saw that FQHC has their own video but they're still trying to work on having it accessible to all staff in the office. 107
- That was a critical component with our patient population. Many of them either didn't have transportation and/or used public transportation or a family member to bring them to the visits. 108
- And for CRC screening we were only utilizing fit kits and then referring out for colonoscopies. So just having patients being able to return that has always been a challenge at FQHC and I know it's also nationally a challenge. 115
- We have a quality team who helps work on sending out notifications on notices. They come out of our corporate offices. In addition to that we have specific little groups, special project groups. For example, we have a FOBT group, so they call for all the FIT testing. We have a mammography group, they help call the registry. And then aside from all of those in our individual clinics, we also have front office and back office doing outreach calling for patients who we haven't seen, patients who have HEDIS. We provide HEDIS lists for patients who have not finished all their HEDIS measures or their care gaps. So, we get those lists, and we can call the patients and cold call to say hey we haven't seen you in a while. Can you come back you're missing your mammography screening; you're missing your cervical cancer screening. So, there's multiple levels to how we reach the patients to make sure they're hitting their HEDIS measures and completing them. 114
- I have the quality team as part of my org which consists of the team that provides all the data metrics and monitoring for our staff on the performance against our quality measures which does include cervical, breast and colon cancer…We also partner with the CRC Registry Team as well. And the Quality Team does a lot of stuff, like many of the outreach strategies for mammo, cervical and… colorectal including mailing out FIT Kits and all

of that from a population health view. And then I also have the health information management team reports to me, which is the team that's basically medical records, which in our system make sure they receive all the outside specialty results and scan them into the chart appropriately. I also have the patient access team and the paneling team, which is buying all our members to clinic and PCP and then also make all the recommendations for how to deal with our access templates, supply demand management and all of that. Which also has to do with we make sure we have in our schedule appropriate blocks for follow up for our patients that are due for screening or our patients that are following up…In 2020 we had transitioned our EMR in August of 2019 from NextGen to Epic, so a lot of the work that was going on at the end of the year to the beginning of the next year was that the transition… we had identified all these screening test results as being areas where we definitely wanted to have them in Epic and not just in the legacy platform that we were using in our charts. So a lot of work on the technical functionality too of how orders and results came through and where they were registering to in the chart…And then for CRC screening we've always done huge campaigns around mailing out FIT kits to our patients, following up on those mailers, doing outreach, reminding patients that they're due for these and it was the same conversation around the transition of our EMR. Making sure everyone was aware where the information was in EPIC, how to access it and that the right information was in there… Nobody likes to take a FIT Kit, they think that's like… definitely always been an ongoing conversations that our clinical teams have had to have. I do recall some confusion around there was a perception at some point that the post office wouldn't accept FIT Kit mailers because of what it was. Which was not true, so there were conversations with teams that that is not true at all, they will absolutely accept this in the mail. But some patients preferred to bring the FIT Kit back into the clinic and how do we make sure we get that to the lab and also make sure the timeline… there's a duration of time that the test is good for, making sure that's all working. I think that was mainly the biggest concern from us and always making sure what's the appropriate follow up as well. 116

- Before the pandemic we were more hands on in the clinic. We would do more tabling events, let's say once a month or every other month we were there providing awareness making sure patients are aware they need to get their annual screenings, whether it's for cervical cancer, breast cancer or just making sure that they're just getting their screenings and answering any [Vendor]ions they may have and then guide them towards asking those [Vendor]ions to their providers. After the pandemic, at the moment we do give them that information as a quick reminder and we try to incorporate it into one of our classes. For example, our diabetes management class, we do cover making sure that the patients are getting all their required lab work or making sure they're getting all their screenings at the appropriate time. So, we do mention it, but we don't go into detail, it's not our main focus. 117
- *I: Way back then pre-pandemic when you guys would do that, did you ever focus specifically on one type of cancer screening, or would you educate on all of them?* R: It was all of them, to be honest. It was just our preventative cancer table, and that was our focus just to make sure that they're getting their CRC screening, their cervical cancer screening, breast cancer screening and just give them that information so that they're aware to make sure I should be getting these screenings already. *I: I am going to jump around between pre- and pandemic and different phases of the pandemic. You mentioned now you do a lot of virtual classes, before the pandemic were you also offering similar types of educational classes, but were they just face to face at the clinic?* R: Yeah so before the pandemic we would just be going to the clinic and providing these in person classes where it was our group classes, same topics, cholesterol, hypertension, diabetes management, our nutrition programs, but they were in person. After the pandemic we switched to remotely and at the moment we only offer remotely virtual classes. – 117

Other Systems (GI specialist/vendors):

- There were sometimes that the lab would run out or they were back ordered with the samples, the kits. So we would have to wait. We would order 100 and they would send us 50 so we'd have to keep track of the patients that we needed to come back to pick up their kit. Right now, since I am not in the clinic setting I don't know if that's still an issue, or if they're good on ordering and receiving the shipment. 107
- Even in general, before Covid, care coordination, that was one of our challenges, where their access was either 1, 2, 3 months out. 112

S3 Appendix: Minimal anonymized qualitative data set

Patients:

- FIT return has always been a challenge. So these are the patients that get it in the clinic, and we're trying to get them to bring it back in some way. When I was in quality we talked to clinics who were trying to strategize or do PDSAs around what if we give the patient a return date? Or what if we scheduled them a visit to drop it off, or what if we provided an incentive for them to drop it off in person they get a gift card. 101
- Barriers of just getting people to go in and complete or getting people to return a FIT test. 102
- So there, in your description there's a little bit of a system challenge pertaining to educating patients on the importance. Trying to figure out not just to do a mass mailing but more focused on the noncompliance. And then of course from the patients, is it fear, is it lack of awareness, whatever it might be. 104
- If the person was going to do a colonoscopy, then that's the whole issue of you have to go into GI consultation. We don't do the prep… consult they do it, the pre-consult. So that's one trip there. Then here's the prep stuff then back to surgical, whatever center it's going to be done and transportation. In some cases, nobody can help me, or guide me, so that's another barrier for some. 104
- So getting people, transportation issues for colonoscopies and stuff so it lets things lean towards IFOB. And then unfortunately as you know people do what they're incentivized to do often monetarily or whatever. We have incentivized or publicized or had people in the back to render, order, order, order. So we'd have hundreds of repeat orders for IFOB just because that was incentivized. Rather than focusing the intention on the follow up. 104
- There was a period where I believe the stool test was actual stool and then they switched it to just the water and we weren't really updated by the lab. And so a lot of the times the patients were doing them wrong, because we assumed it was still the stool sample not the water sample. 107
- Here and there we'd have some patients that didn't want to, because they didn't want to touch how they say their poop. They said they felt fine that they didn't want to do it. We had to keep asking and reminding them. 107
- I would say at least with colorectal cancer screening they've always been reluctant to do it, because of the nature of it. The inconvenience of having to prepare for it, bowel prep takes a lot of time and effort for a lot of patients. 110
- FQHC primarily services low-income areas with high health disparities so getting patients to come in for anything has always been traditionally very hard for us. 115
- Colorectal cancer screening is always challenging. I don't think much of that has really changed, pre- or post- but it's to do because of a couple reasons. One was there's a large number of patients that need it. The other part is the key of trying to get patients to actually do the test. So those challenges still remain, and that was one of the challenges.105
- Prior to Covid it was great, FQHC has really high rates for doing our HEDIS and cancer prevention screenings. I will tell you since I've been there FIT testing is always the one that the majority of the clinics struggle with the most. We've partnered with Kaiser before, we've used outside agencies to try and get the patients in for colonoscopy and FIT testing. That's always been the measure that's the most difficult to meet our HEDIS marks. *I: What's your sense of why mammography is easier or why they were able to get their cervical rates up but FIT is a challenge. Do you have any observations of what's causing that*? I think because patients in particular, breast cancer there's so much focus on it. Celebrities, we do Revlon, Koman Foundation. I think there's so much PR and marketing around breast cancers that that one is very tangible and in the forefront of people's minds. The same as probably getting a pap and cervical cancer. I think a lot of people know somebody who has had cervical or ovarian cancer. I don't think it's promoted as much as breast cancer but I think it's promoted more than FIT testing. I think with FIT testing there are some reservations because how we do it, people are like you want me to do what. There's reservation because of how we test for it. Colonoscopy, I think there's still to this day not a lot of education on it. So I think people in their mind are thinking what you're going to put something up my butt for lack of better nomenclature. I think in the male Hispanic population that's a big thing. And I've had patients tell me no I am not gay. No, that's not going to up there. I am not gay. Well it has nothing to do with you being gay. And I think there's hesitancy in the older Latino population for that, just from my own observation. So I think that's probably a lot to do with it. I think there's just far more information, education and a bright light shining on breast cancer. It makes sense that that would be the most popular one people agree to do. 114

- So I think it was once we were able to provide that information or that education on the topic, they were more aware to talk to their providers. That's something that once we had that tabling, we would mention it, talk to your doctor, make sure that he… that you let them know that you wanted to get these tests done. So that's something that we try to encourage them to just ease up that topic, because I know that especially with our clinic, where our population is, there's a lot of Hispanics that don't want to talk about those topics. They're like oh no I don't want to talk about this. I don't want to open that topic. So there's some patients that feel embarrassed to talk about those topics. Even when we had those table events at the clinics, a lot of patients would skip through our tables, they didn't want to talk to us. They felt embarrassed talking about it. 117
- *I: Great. In terms of that assistance you were describing you provide, is that for all the cancer screening services, like colorectal cancer, breast and cervical? Or do you focus more on a particular cancer screening service that you support?* R: Regarding those cancer screenings, normally if the provider messaged me to give them a call to schedule, I will go ahead and schedule them. I focus more on forms that the patients bring in like disability forms or B&B forms. I am more focused on referrals from any specialty that they're being placed. As far as the cancer screenings, usually on Saturdays if they're coming in to see urgent care, I'll go ahead and set them up to have those preventatives done that same day. I am not familiar as like getting the list of the patients to call them and get an appointment scheduled for them. We do have those registries that they give them a call. But if the providers send us a message then I go ahead and schedule them as well…For colorectal I send them the kit at home and I call them and let them know I went ahead and sent you the FOBT Kit, or Fit Kit. If they already had one done I schedule an appointment to see the doctor. Or if they have a referral to see the GI for a colonoscopy I try to assist them to schedule an appointment with the GI so they can have that exam done… I get a message saying they went to go have a colonoscopy done and then the resort got sent to the provider, usually the provider will send me a message or their provider partner a message to schedule the patient for a follow up to go over the results. Any further elaboration that's needed, I still assist the provider to scheduling those appointments. 119

Other:

- We had the same issue with the colonoscopy. That was almost all colonoscopies. We don't do any colonoscopies through interface, those are all specialists. So that we're looking forward to getting that fixed. That may have a big impact on our performance. So all my anger at the FIT kits may dissolve a little bit once we get that data issue fixed. And that data issue just to clarify, the data issue I am referencing dates back to when we switched EHRs. So that had been in place pre-Covid, through Covid and now post-Covid it's being fixed. I think that illustrates another challenge that we've had through Covid. 102

<u>CRC cancer screening facilitators (or things in the works just before pandemic)</u>

FQHC:

- We actually developed a contract with Safe Ride. And Safe Ride is a medical transportation 3 party who utilizes Lyft and Uber services and they contract with different health plans, urgent cares, hospitals. Typically the patients could use up to 4 rides for free, anything after that could be charged to the clinic. We would work with health plans, many of the health plans, for example CalOptima actually did have some transportation services built into the patient's overall coverage. 108

Patients:

- I think just education for the patients. For a while it was just us explaining to the patients how it's done. And then they were able to program a video on the FQHC website and we would show that to the patients. That helped a lot for them so they could understand visually how it's done. 107

<u>**Early COVID Phase (March-July'20)**</u>

<u>CRC Cancer screening barriers</u>

FQHC:

- I think the biggest impact was that we had... less visits and I don't remember when exactly this happened, but there was a goal, I think that was a few months in, June and July, to convert a lot of the in person visits, and I think the goal was 40% of them to tele-visits…But then also hard to give a FIT kit at point of care, so the FIT mailing still happened that year. 101
- Quality. So we know that the FIT order was being placed FOTB order for colorectal cancer screening, and then the [Vendor]ion was okay well they have the order. Traditionally we'd give it to them in person but who does that now. And so it was difficult to resource like oh actually and eventually this was the directive that came down. Yes you place the order and someone in your back office has to mail them their AVS, because that also includes information on any referrals and orders that they had aside from getting the fit test and also mail the FIT test. So that's now been centralized but at the time it fell back to the clinics to do it on their own. 101
- Can I ask a [Vendor]ion? Part of that was so a provider might've been working from home doing tele-health and placing that order for the FIT and then it was required that someone who was on site in the back office would have to do all those other steps. Yes. 101
- So during that particular time we did not have many, if any Covid, any type of cancer screenings unless there were those who had an urgent need, prior screening right before the height hit and needed clarification scans or screenings. Those we would continue to see or refer out to an open facility. 108
- Colorectal cancer screening, colonoscopy centers, the GI centers were closed because of the precautions for Covid. So that was an access issue. We did have the FIT kit mailing part but we were really in crisis mode here trying to adapt to all the changes. So although we were giving out FIT kits at the offices there was no big mail in campaign at that time to assist with that so we were behind on that for the same reasons again. 105
- It's a heavy inbound queue, and outreach is really done off queue, it's off queue work. So one of the unfortunately when Covid hit we were so busy and overwhelmed with the amount of calls coming in through our general line that for outreach we had to actually pause our outreach campaign. 112
- R: It started off as organic and then it became preventative, that's goes on the back burner right now, because honestly we were so inundated with testing and Covid patients that everything just went on the back burner. And then on top of that we were losing staff left and right because they were getting Covid. So we had less staff, less providers at the clinic and most of our focus was like I said to urgent episodic visits versus any preventative or maintenance. And then it came to the point well none of the kids were in school, so no one needed physicals. And hardly anybody was working because lots of places shut down, so we weren't doing work physicals either where we would've had the opportunity to be doing screenings. Even our MHAs that we do for the health plan, all of that was halted. They halted those and we were told not to do those screenings. We didn't want to bring the frail elderly to the clinic with all the sick people. I take it you guys weren't doing any calling, come do your FIT tests? R: No, we didn't want patients at the clinic at that point in time, because we didn't want them exposed. That was still that period in time where we're like we're not really sure how this spread. and we didn't have PPE for patients, we didn't even have PPE for ourselves. So how were we going to protect them. So the ask was from our leadership let's not bring preventative in right now…. I lost staff who got pulled into doing what we called Covid calls, because we were doing so much tele-health. So I had staff pulled for that. We had staff pulled to some of the testing centers, like the main testing centers….Now FIT testing, people were just not... we were doing the mailers and everything. Mailing them out, people were just not sending them back. They just wouldn't come in. One was because a lot of them we weren't letting into the building. Why are you here? No, you cannot bring 4 people in, we only let the patient in, you have to have a mask. So that became a barrier I think. *I: Was that a barrier to returning a completed kit you mean?* R:Yeah returning the kids, if they got them in the mail, having them return them, we would tell them oh you can mail them. But for some reason they just don't like to mail them. They like to bring them into the clinic, so that was a barrier because we wouldn't bring them back. It was a combination of multiple factors. *I: Going back to colorectal and FIT tests that interesting about people getting them in the mail and then wanting to drop them off at the clinic but then not being able to if they didn't have a mask or couldn't come into the clinic*. R: Or did not want to wear a mask. We had the whole front of the building roped off. So even to this day you have to be screened. We take your temperature, screen you and give you a mask to be able to come into the building. You can't unless you have all those things done. And for some people they were hesitant. It's amazing how many people think that if we take a temperature on their forehead it does something to their brain, so they would refuse and say no you're taking my temperature through my forehead. But uh, that's how we have to do it! And so they wouldn't come in. They'd be like nope. Okay, I guess hand me your kit. It was really weird barriers, things like that, little nuances we had to work around. 114

- I think one of the things that impacted us greatly was… our physical space in our clinics we don't have big foot prints. A lot of our clinic spaces are in locations… some of the older clinics are in older building and sites. So over time there have been basically a lot more people closer to each other in that space than what you would require now for safe space constraints. We walked through all of our clinics and blocked out the ability to use some of our rooms so that we could make sure people could maintain social distancing. That meant that also even the ability to do things like *paps, just* meant we needed designated space that was slightly bigger too. And so that limited our capacity which made it more problematic. I think the same with CRC, FIT Kits… We were using FIT Kits and also continuing with the FIT Kit strategy because many of the outpatient procedure centers weren't doing colonoscopies, at least for screening. 116

Other Systems (GI specialist/vendors):

- It really depended on the facility, many would take days and now weeks, and in some cases specialists were not available at all. But certainly exacerbated that time frame from days to weeks and in some cases it could've been months if it was something we're looking for in clinic. Outside of that they would go to a hospital. 108
- I would say very unique, urgent cases would go and get it done, I remember. But a routine type of screening like the way we do it now was pretty much stopped because of doctors... not wanting to see anybody unless it was an urgency, an urgent matter. Especially a colonoscopy who is not immediately necessary I believe was postponed for a later time. 110
- We still refer patients out no matter what was happening. But it was just a matter of a specialist being able to see patients. 110
- Patients had their colonoscopies scheduled for some time then they got canceled. And a lot of those patients lost track of going back, calling their doctors back and say it's been a whole year, can I get my colonoscopy now? It's like it was forgotten. I don't know what the effort for GI was to call their patients back. I've heard patients say I called the doctor, but they never called me back. It was canceled and I never heard from him. 110
- There was definitely a delay, a few months actually. We have data that we looked at and there is definitely 3-4 months of delay. Because they couldn't do the services. And honestly we are already challenged because of our patient population and their insurance. A lot of our patients are MediCal, so we have a limited network availability and then those offices were closed down, it was really an unfortunate set of circumstances. 105
- *I: How would you say it unfolded, the FIT kits are probably offered during an office visit, so once people were starting to come back in, to actually be seen face to face by a provider, were there any challenges in trying to reinitiate making sure someone goes home with a FIT after an office visit?*
  R: Yeah because I will tell you the other issue we had, and another caveat was we could not get supplies. Often times the supply chain got broken. [Vendor] which is the majority of where we get our FIT kits, they were so inundated with Covid swabs and Covid supplies and doing all that, there was a while we were limited on how many FIT kits they would give us. Normally we order a giant case, and I take them home and label them, I pre-label them so the patients don't even have to do that. And they would send us a box or two at a time. And we're like no, no, we ordered a case! 40 boxes of them. They limited supplies to us, that was a big thing too, [Vendor] and [Vendor] limited supplies for a while. *I: Do you recollect when that was?* R: 2020, yeah. A little bit into 2021... it's kind of ebb and flowed, because even still to this day we're still having supply chain issues. Like we can't get certain lab tubes, like vacutainers. So it's ebb and flowed, right now we're still not getting the amount of FIT kits that we normally re[Vendor] from them. *I:Do you have a sense it's because they're still backlogged because they're...* R: Trying to catch up. They told us a lot from the suppliers, who [Vendor] gets their supplies from. They would tell us hey they're not even coming to us. We can't give them to you because they're not coming to us. 114
- *I: How about in terms of, I don't know how much your staff is involved in this. But obviously when someone has an abnormal fit then there's a need to try to get them to GI for a follow up colonoscopy. Are you involved in helping to facilitate that and how did that get impacted with Covid?* R: We have something called care gap coordinators in the clinics, CGCs. So when we get abnormals the workflow is that the provider lets the patient know. Most of the time we were giving abnormal results via telephone. We didn't make them come back into the clinic, we would do a telephone visit with them. And we would try and get them scheduled to one of the groups depending on their insurance. But even that we couldn't get in. And at one point in time our referrals were backed up 6-8 weeks even to get the referrals processed. So we'd have that backlog to get the referrals

processed through the different agencies, like Cal Optima or Anthem or anybody. They were so backed up. And then by the time we would get it processed and get it off and get an approval, then to actually get the patient into the Center was taking 3-6 weeks. So there was delay after delay after delay outside of our walls that we couldn't even control. Same thing... didn't have staff. *I: When did that start getting better?* 2022. It started getting better not until 2022. Probably early spring 2022, where it's starting to get better. Every now and then it will get backlogged again. Until probably... we're in July now, up until May we were still having 2 months turnarounds for getting off approvals. That's how backed up the insurance companies were just to get our referrals even approved. We have a whole team that works out of corporate to help with that. A whole referrals team. In fact they pulled some of my CGCs who were really good to that team in April to try and help facilitate to get all these off, because now all the patients were coming back and were like referral, referral because we haven't seen them. So imagine we tripled the amount of referrals we were trying to process. So we literally put together a whole team for that. Like a management team. 114

Patients:

- Now looking back, because now we have that... now we're look at all the patients who were abnormal at one time. And some of the conversations in the notes say will follow up post-Covid. Will get a colonoscopy scheduled post-Covid. Waiting to see if Covid situation changes. So clearly, and I wasn't having these conversations with clinics, but clearly patients were waiting to see if things got better before they followed up. 101
- Maybe FIT kits a new thing that came up because of the lock down they weren't living at their home, so we mailed it to their home. Well, they haven't been at home for 3 weeks. So there were some difficulties that came up with that that we hadn't foreseen before, a lot of people moving around, a lot of people not wanting to go out to their mailbox. 102
- Yeah so I actually had a couple with positive FOBTs and it was again a risk benefit discussion with them of what's kind of… what is their comfort level, which way to go. So worried about getting Covid and possibly dying that they want to defer this. And I tried to help them give reasonable estimates the best that I could. I monitored the statistics and hospitalization stuff trying to figure out ballpark what that would be for a person 67 years old with blah, blah. And in a few cases ended up repeating the FOB knowing it's not the best but it provided some potential reassurance. 104
- Just that a lot of patients would not go to the clinic. They were just afraid to go into the clinic because they thought they would get Covid right away. 107
- Many of our patients just didn't feel comfortable leaving the house at all. 108
- Transportation is always an issue for older patients. Transportation, not having insurance to pay for it. Financial hardship. 110
- Once we implemented tele-health that was the First time we actually started mailing kits out. Before that they came face to face and picked it up at their provider visit. But with the implementation of tele-health with the provider, that's when they started mailing them out to patients. And I know our back office really tried to get the patients to mail it back to us, so we were reducing traffic in the clinic. But we've also had patients not trust the postal system or they want to come and physically drop it off in the clinic. So if that was a barrier for them they just weren't going to do it. 118
- Absolutely, there was definitely a delay there as well too. That was not a focus area for them. And they were afraid, they didn't want to go to facilities also. Screening is something we always have to have discussions anyway to encourage patients and that was not easy to do. 105

CRC cancer screening facilitators (or things in the works just before pandemic)

FQHC:

- They have the care gaps, it's kind of like HEDIS and it shows what the patient is due, whether it's just for colorectal or fecal test, or mammo or pap whatever it is. If we see that they were due for their stool test, their fecal, we would let the patient know and we would mail out the kit for them to their home. 107

- I guess that's how the registry was born. It was more... we won this CDC grant but I guess we were... I believe struggling to have a good system to be able to refer patients and track them, so we could assist them better. 110

Telehealth

Description:

- Wow, well there was kind of like a command center, where we were supposed to be doing that. And then sort of sprung up telehealth center, a call center, so we could see patients if we were totally remote and seeing patients there was a very special room for us and then the discharge side we had to send a message to some pool there to follow up with a patient to go over next steps, scheduling for stuff and sending their AVS. But that from my recollection there was a command center, it was frenetic at that time. A lot of providers just shifted over. 104
- In the beginning of tele-health during the pandemic it was chaos. We didn't know how to navigate. 107
- March... of 2020, probably by April of 2020 we were 80% tele-health and 20% face to face, so really rapidly we converted almost all of our providers. I want to say they were doing 4 days tele-health and one day in the clinic. So it was a pretty rapid change. But FQHC implemented it very quickly. So yeah I want say within that first month it was converted to 80% tele-health 20% face to face if not more than that. 118
- we were now doing 60% of our visits via tele-health. They used to be zero before, they used to be face to face visits where we could at least get something done when they came into the office.105
- Before the pandemic we did not have any telehealth at all. Some providers would call patients to talk about their results but we didn't have a way to bill for it. So it was essentially a virtual visit, without it being an official visit. Then once the pandemic hit and the state of emergency came out we were able to do telehealth. We started the telehealth program out of our patient service center. 111

Barriers:

- One thing that came up was that, this is even before Covid, we have a pretty difficult time with patients to do their FIT test and do it wrong. Do incorrect collection. The clinics have always said having them in person and being able to demonstrate how you brush the stool, what you do after, how you put it on the card is central to having lower error rates. And so obviously that's gone in tele-health, the provider is not doing that. 101
- And colorectal would be a FIT kit. Now that was a challenge because what we found out after the fact was that a lot of orders were being placed for FIT kits for patient but nobody was sending the FIT kit. So then when my team would do the outreach and say hey you had a FIT kit mailed to you. No I didn't, okay we'll mail you one. So that's a gap in our tele-health process is they would order the FIT kit and FIT kit wouldn't get mailed. 102
- A lot of the patients, especially the older population they didn't want it because they wanted to physically be seen by the provider and they kept saying that they wanted to have their vitals taken in person and have the provider listen to their lungs and their heart. And some of them didn't know how to navigate through the phone if it was a video or telephone visit. 107
- With our specific population of patients it was definitely a challenge to get them to do tele-health, a vast majority of our patients were willing to wait to see a provider face to face. And in that time it was months to see somebody face to face, because they didn't have the technology. They didn't have the knowledge of how to download the application or a lot of our patients too, mostly 90-95% Spanish-speaking Latinos in this clinic. And they just didn't feel comfortable talking to a doctor over the phone. 118
- Yes, that was still on site, up to 40% of our patients came in, but 60% of patients were on tele-health. And then we had the challenges of tele-health too. There's different ways of providing tele-health, one is telephone. Which is not the preferred method, the recommendation was to do more audio visual but our patients were challenged with the visual part, they didn't have very robust internet connections, their phone services couldn't handle it. So a lot of it more was telephone than audio visual. So a lot of the providers had to do more telephone visits or audio only visits, other than audio visual visits. So although we have audio visual visits even now available that has still continued to be a challenge.105
- There was no tele-health at all before that in our health system. Which is not unusual, it was like that in many parts of the country because it was not a covered benefit on our health plan. And then there was also the issue at the state level that there was no assurance that this would be covered at the FQHCs. So there would have to

be a lot of work done to actually get that approved for the FQHCs. So those were the two barriers with tele-health.105

- See separate telehealth report for 114

Facilitators:

- In my site we didn't mail kits, we would usually give the patients in the office when they would show up to their appointments. Later, when they did the tele-health visits, that's when they started mailing out the kits. 107

Other impacts on Care at FQHC (staffing, closures, shortages, redeployment, focus of work, PPE, communication, etc.)

Health system operations:

- This department would hold a lot of outreach attempts and run a very fluid outreach program, in conjunction with our call center, with other grant teams, within our organization. And when Covid started that was a very big part of this department that was impacted. So we weren't sending out fliers to patients anymore, because of the low bandwidth and a lot of limited access within the team we weren't even able to do outreach attempts with patients like calling and texting anymore. And the same with the call center because they were so impacted by how many patients were calling in and trying to schedule an appointment or trying to come in for a Covid test. So all of that was halted for about 2 years. 115
- Yes everything at that time was shut down, and staff was furloughed because of no work or sometimes let go. So there was also staffing that started to occur at that time, it was the beginning of that. 105
- There was lots of staff impact, lot of movement. There was definitely redeploying of staff because we had to stand up our Covid testing sites so that was one thing we did a lot of initiative on and helped with. But we also had... there was furloughs of staff. Some staff left on their own or they were let go because we had to relook at finances so that we could continue to keep the operations going because of the huge impact overall. So basically yes, all of these staffing issues occurred. And we were definitely with left with less staff in the end. 105

Changes to clinic staffing, redeployment, PPE, work from home:

- Staff-wise it was kind of hard. The workers were focusing on face-to-face patient care. And then helping the Covid patients so it would split the clinic, it sounds harsh but sick side and not sick side. So it made it harder for us to have the staff and to explain to the patient and to be on top of the tests. 107
- So I think one of the barriers in terms of access to care is that we certainly had to reevaluate our staffing plans. So one was our corporate offices are in cubicles that weren't... spaced, weren't necessarily closed off. So there was a huge adjustment there, but then also within our clinics, so staffing was significantly reduced in our clinics because we needed safe space. Our front office, our back office, our providers needed to sit 6' apart. Our lobbies were closed. We couldn't have patients sitting in the lobby because we didn't know if they were ill, if they were healthy and we couldn't risk the chance of infection. 108
- Additionally, we had staff that was out sick…At that time there were 10-day, 14-day mandatory quarantine, testing. We had a lot of our corporate staff redeployed or our non-clinical facing staff redeployed to different areas that needed to continue on but had to do remote work. Additionally, we redeployed a lot of our front and back office staff from various clinics to two large hubs. We had a Covid testing site in Anaheim for our Orange County location. And we had another at our Goodrich location in LA. And we were testing well in excess of 3-400 patients a day. So we redeployed to those sites to maintain that demand. 108
- For that specific time period of March to July 2022, my knowledge is that most of the corporate offices were redeployed into serving the call center. And all HEDIS measures like cancer screenings were put on pause. 115

Mid COVID Phase (July-2020-Dec 2020)

CRC cancer screening barriers (for each cancer)

FQHC:

- And the only issue is we would get a lot of back orders from the kits. A lot of the labs were back ordered. I don't know if it was true but they said hospitals were priority for everything. 107
- Them being able to schedule either with a provider or specialist office for continued care in case their test did come back abnormal. 107

Other Systems (GI specialist/vendors):

- I think it's just like capacity. I am not sure what it was before then, but even now if a patient has an abnormal test, they're probably not going to get a consult until May. That means they probably won't get their colonoscopy until June. So we know the continuum from abnormal to getting the test can take months. And that's for a delay initially getting the patient in, but also I think another barrier is that they want the patient to get a Covid... test before they get their colonoscopy, so that creates an additional step. It's a procedure the patient probably doesn't want to get either way. 101
- By 2020 the majority of the patients didn't care. Even the diabetic follow ups. If they had blood pressure, we had patients that were going a couple months without their medication because they were just afraid of leaving their house. As far as I knew we didn't have resources to help cover, if it came back abnormal the fecal test we would refer them out to a low cost clinic and I believe their appointments were far out too. And they didn't really offer those services. They had to refer them out somewhere else as well. So this patient still had to pay for their test but I guess it was lower in cost. Patients felt it was hard for them to be able to pay $800, $1000. 107
- Yeah there was a list of gastroenterologists. I work in Orange County so it was specialists in Orange County that would accept cash patients but we didn't know actual prices for the consult and the procedure. So we would hand it to the patient let them know they should call and ask the prices and see if it's something they're able to afford. If not what are the other low income clinics. A lot of time patients didn't follow through, it was too expensive and time consuming. Now I know we have an updated list with gastroenterologists in both Orange County and LA County that tells them the prices and provider. 107
- Really delayed because a lot of the providers didn't want to do face-to-face consultations. Some very rarely would do over the phone consultations. And I believe the ones that needed to do hospital settings, they had a lot of restrictions and they had to wait for them to be clear to do the colonoscopies in the hospital. So some were waiting 6 months or more just to have a consult. 107
- We were still seeing the long delays with specialties. There were so many specialties that were completely closed down at that time. And the ones that stayed open, the waits, it was months to see a specialist at that point. Because they would only take a few patients a day. 118
- I think the only other thought I have for colonoscopies, those were few and far between, just because of the challenges of patients would need to get tested before they actually went in to do the procedure. And a lot of places weren't doing preventative procedures or elective procedures so to say. And they were holding off on those and only doing surgical cases that were necessary. I can only imagine that the numbers were driven down significantly for colonoscopies. 111

Patients:

- In my site we were more comfortable with it. It was just a little bit overwhelming sometimes, we would have to call patients for 2 or 3 different providers at the same time. And all the patients were scheduled 15 minutes apart. I can't remember, I think it's still the same. So we would call one patient and if they don't answer then we would call another patient and then we would have the other patient returning our call and we're already on the line with another patient. So it was a lot of playing phone tag. 107
- I think it was the overall adjustment as well. So now in some cases they weren't necessarily fearful of coming or leaving the house, but they would approach the clinic and things are different. They're being scanned outside, they're being asked to wait in their cars. Some of our patients didn't understand that process and then it was a little difficult if I came on the bus. 108
- Patients didn't want to go out of their homes and see a doctor for a colonoscopy. 110

CRC cancer screening facilitators (or things in the works just before pandemic)

FQHC:

- We would mention if they would bring back the envelope with the sample that we would give them a gift card. Our site was providing $5 gift cards from Target. If they physically dropped off the form, they didn't even have to come in person, they were able to go through the drive through and hand it to us. And then we would provide them the gift card. A lot of patients when they heard okay I'll get something in return if I do this, they would do that. 107
- In relation to access and availability and ease of processing. It would've been our colorectal scheduling. Our teams had a great focus where we were able to mail out Fit kits and mail out the screening materials to our patients. They could take it from home and mail it back. 108
- Meetings and huddles and we actually added a new parameter. We created dash boards so we could actually track it a little more closely and we focused specifically on our cancer screenings. We had our data team develop dashboards to see what our denominator was in relation to patients who needed screenings. 108
- Not only did we reopen but we now began to develop workflows with our quality team and our call center team to begin active outreach for screening, for all of them, for mammograms, cervical, colorectal. 108
- The benefit was we stopped sending bills throughout this entire time, so all of our care virtually at this point was free. We just started resuming the transportation services assistance in the middle of 2021. 108
- Now colorectal cancer, we were as creative as we could be! [laughs] The main one was FIT kits, we did FIT kit campaigns. So one we partnered with... we have a grant for colorectal cancer screening with FIT kits. We have a registry for abnormal FIT kits there. That team helped us with this endeavor. So we told our providers again when they were on a tele-health visit to go ahead and place the order for FIT kits during the visit. And the registry team would then take it upon themselves to mail it out to the patient. So that way the patient would get it at home. It would be all filled out so they could just mail it back in. So we tried to take out all the barriers of not wanting to go into the facility to do that. 105
- CRC screening, I don't know number wise but we were mailing out those kits to patients at their house. So overall that shouldn't have had much of a difference, unless there were more addresses that weren't updated so they weren't getting to the right house. 111

Other Systems (GI specialist/vendors*)*:

- I believe they had opened up a little more, particularly colonoscopies is what I am thinking of. 104
- We were still backlogged in some regards in certain areas, referrals was definitely one. 108
- And colon cancer screenings, same issues with colonoscopy, access was very limited. Again they had a backlog and then it took months to get them in. We were able to continue to do the FIT kits. 105

Telehealth

Barriers:

- The way I recall it was about the same. We kind of hit our stride. There's way more uptick on telephone than video. So we had been frenetic on the informatics side trying to get a solution for video visits and they actually had 3 flavors of it for various reasons. But then there wasn't so much uptake on that. And interestingly if you diagnose something during the telephone call for Medicare patient, like diabetes with renal complications, that diagnosis doesn't count in terms of the ATC and all that coding stuff with the health plan we work with. So that came to light and so there was crazy things. Don't allow phone visits for Medicare patients. No we can't do that! They're the population that probably can't use the video stuff! 104
- That's another challenge, providers that don't speak Spanish you have to get a interpreter to join which is difficult to do. 104
- A lot of it too you see a video visit with a lot of patients their internet is not great. So the quality is bad and unstable internet. And on our side it's helped us beef up all of our WiFi and internet systems and at times to have to face outside to do screening and stuff. So we've learned a lot and I think there is a little bit of a silver lining in this thing. 104

S3 Appendix: Minimal anonymized qualitative data set

- I think the biggest adjustment for our staff was the tele-health. Going from face-to-face visits, now having virtual care, virtual visits, there was a workflow change, there was a system change. Processing and building those relationships with patients over the phone, that was something we were adapting to and to some degree still are. Getting patients to have visits via video versus telephone. Making sure they have WiFi. 108
- Actually that's been a huge help, and we still encourage patients to see providers via tele-health. And that's one of the barriers I have encountered with GI specialists that they are not doing it. I mean since last year when I started with the registry, I haven't really seen it happening very much. It's almost like they want to see patients in person and they don't offer this telephone appointments as much as I would want them to do it. Just for consultation I feel like they could do more telephone appointments. 110

Facilitators:

- FQHC primarily services low-income areas with high health disparities so getting patients to come in for anything has always been traditionally very hard for us. That's why tele-health was a great option for those patients. 115
- During the reopening phase we prioritized women's health in particular. CRC can really be done telephonically because we do the at home FIT kits at Alta-Med. So that was a good transition point for CRC screening. 106

Other impacts on Care at FQHC (staffing, closures, shortages, redeployment, focus of work, PPE, communication, etc.)

Communication re protocols or changes in protocols:

- At my site we would have clinic meetings or huddles. Either our supervisor or our clinic administrator would let us know the process and about the gift cards. 107

Changes to clinic staffing, redeployment, PPE, work from home:

- When they were abnormal, then the referral process would kick in and sometimes that would delay some of the responses staff-wise because there were some people that were let go with the company. So I know a lot of the departments were backed up in their work. 107
- Definitely staffing, that's when staffing really became an issue. They, we just didn't have a lot of staff. I think it was due to other factors but Covid made it so much worse that we were really short on staff. I know the company had to let go of certain people or furlough them. So staffing was definitely an issue. All of our providers stayed on but we just didn't have the back office support. And the front office, to be honest our front office was pretty short as well. 118

Later COVID Phase (2021)

CRC cancer screening barriers

1. **FQHC:**

- Part of the team now follows up on FIT tests that were ordered. And I know at the time that they were calling to follow up on the patient. 101
- We just had a challenge thrown at us with colorectal cancer screening, now it's opened to 45-49 so we just added 15,000 people to our denominator. And our data shows that typically the 50–60-year-olds are the least compliant. So we are again regrouping to say we already know the younger population doesn't does this, how do we get the even younger population to do it. 102
- I just think in general there's not that much information to educate and alert the patients that they need these screenings. On site, I don't really see that many posters or commercials, explaining to them why they need it and why it should be done. A lot of those times it's just us in the back office with the provider telling the patient you're due for this you need to get this done. 107
- The clinics are still very impacted not only from Covid but in general with the great resignation, we saw limited staffing. So a lot of the onus falls on to the corporate team to try to encourage patients to come back to the clinics. For example, my team has finally after 3 years, 2019 was the last time we did it. After 3 years we're

finally picking up our breast cancer awareness campaign again. And that's going to be outreaching to all of our patients who haven't had a screening done during the Covid times. 115

- We just started resuming the transportation services assistance in the middle of 2021. 108

Other Systems (GI specialist/vendors):

- I didn't have an idea of the delay in gastroenterology scheduling until this role. So we saw a lot of patients who had an abnormal who never followed up. That's actually been the case historically but even more so during that time who had an abnormal FIT test and couldn't get an appointment or was many months out. And just like a backlog. So once we launched the team in April of that following year 2021, there was a backlog of 300-400 patients who hadn't taken action on their abnormal test. 101
- So we use [Vendor], not that we heard of. Some of our lab things to [Vendor], FOBT is not up there, they do way more. CBC is the number one order. So they have other top priority tests before maybe we'd be impacted. But yeah no delay. Maybe one with mail, but I don't know if that was related to Covid or just USPS problems. 101
- And then offices were also opening up more so they were able to schedule with the specialist, surgeries and the procedures that needed to be done. In 2021 it was a little bit easier for them to actually do the consult. I think it went from 6 months to 1-3 months and the procedures are done fairly quick as well after that. 107
- The new barrier was now the requirement of vaccination for certain services. So even if we were doing the screenings, if the patients weren't getting vaccinated some of the specialists wouldn't see them. 108

Patients:

- I think by this time, it was a little more difficult because they had to prove negative tests and stuff, so many days before the procedure. Not for mammograms but for colonoscopy. 104
- I think the largest challenge was resuming normal health care for them. And what I mean by normal healthcare is encouraging the patients to come in for screenings. And this is still a challenge now, it's not just for their screenings but their patients are just coming in to get their medication refilled. And that's still a constant problem, so how do we change that culture back into coming and getting those screenings done. 115
- I think in 2021 sit was we know we have a lot of catch up to do and we need to get on this right now in terms of the people that we know are due. Because I think a lot of our patients self-selected. Everything shut down, and then… and a lot of people just… weren't accessing like they normally would but in 2021 it was like we wanted to… we were doing huge outreach, number one for the vaccines. Number two for the vaccine was also an opportunity to reengage patients. We're so glad you got the vaccine, this is great. And you're also due for XYZ. We also were able to get some technology to help us with texting patients and optimizing our… since we had transitioned to Epic, we had My Chart. So that was great because we could leverage all the self-scheduling functions in My Chart, we could send messages via that way. And that's really been where we've focused now, certainly is how do we leverage automation as much as possible so we can engage patients in their health and letting them know what's going on. Understanding that everybody's got a very busy life and making it easy for them to get in to see us and know when we have availability for those things. The registry teams we increased the size of too because we knew we needed to get more of their support. And if we were doing more testing or follow up at once we would definitely need more of our LVN staff to help us making sure, monitoring and tracking through the whole process of any abnormal findings too. The good thing of Covid is some of our services we centralized and that made it a lot easier for us to see what is going on throughout our total care system. So during Covid we centralized all of our coordinators that enter referrals into our authorization platform to our UM team. And that was great because I had one team that I knew I could go to and who also could partner with our registry teams to make sure hey I got the opp, I submitted it, ball is in your court, that other centralized team who could then follow it through and then work with my team if they had any issues where they had to close the loop with the specialist or that office didn't have the best access can we go to another one, all of those processes… I feel like we had a bigger surge, it was interesting in terms of general referral volume. I don't know how many of those were for cancer screenings. But it was the last quarter of 2021, where it was like okay we're seeing greater volume, and part of that too was a lot of the specialty offices also hadn't opened fully back up. So that was managing the connection with all of our contracting offices too to make sure we understood who was fully open, who wasn't where we needed more help and all of that too. I feel it was more mid- to late-2021 where we were seeing the downstream impacts. 116

- *I: When would you say that shifted or changed for patients, where maybe they became more willing to consider... Oh yeah I haven't gotten my FIT test done in a while, I'll go ahead and do that. When did you start to notice a shift, or maybe you haven't?* R: When California reopened again we started to see more fluctuation of patients coming in. We were starting to open a little more slots of face to face with the patients. And we were starting to see a little more optimistically with the patients as far as them wanting to do more preventative. And then we shut down again and they got scared. So our numbers went down a little bit. 119

CRC cancer screening facilitators (or things in the works just before pandemic)

FQHC:

- This is part of the work that's supported by the CDC grant. It's LVN, RN at the time that basically helps schedule on behalf of patients. And so they'd reach out to patients, convince them to follow up, schedule if needed, have a direct line to our GI practices. We also eventually had an ability to financially assist them... that's actually been the case even pre-Covid if they were uninsured, and they didn't have options if they had an abnormal test. 101
- We had a lot of patients, the majority of patients that were due for their actual screening. So once they were in the office we would take advantage of all that. Our site would have poop emoji plush toys all over the clinic and it would have a sign asking how you can receive a gift card. So it would catch the patient's attention and they would ask us and we'd tell them you have to do the fecal test, you receive a gift card. 107
- I feel because the registry was in place, and we were able to track them... call them the first time, I would call them, give them their results, tell them about their referral. If they needed anything, if there were any barriers we were still able to help them. If they don't have transportation we can provide them with transportation. That's not an issue. If a lot of them don't have insurance and they can't pay for it, we also help them pay for the consultation and the procedure. If they needed appointments because they couldn't get through because they couldn't call or whatever reason we are still making appointments for them. Yeah I mean we would try to help them with as much as we can. 110
- FQHC has a program called Gives and they have given us... money to help specific patients in the CRC registry to get their colonoscopies. And it helped about 20 of them already. And we keep getting more money so it seems like we're not running out of money so we keep getting $5000, $10,000 here. So yeah we're able to help patients. We encourage them to look into obtaining coverage. And if all fails we help them. But we do encourage them to get, especially because now Medi-Cal, the new medical expansion and how a lot of those people that had Medi-Cal and emergency Medi-Cal now are able to get a more complete Medi-Cal and they can see their specialist and all that. FQHC has put in great, great effort in trying to help all those people that did have emergency Medi-Cal or they're 50 years old and above to get coverage, real coverage. 110
- And then for the CRC screening it continued to be, a lot of patients were sent out their FOTBs their FIT kits to complete and bring them back. We definitely saw an improvement in them coming back into the clinic. 118
- We have created registries. I don't know if we could attribute it to a product of the pandemic. But we created registries that were helping to follow the HEDIS measures and need for follow up. 111
- What we have done to offset some of that is our quality team is actually focusing on the colorectal screening. In Alta-Med we call that a FIT kit test. We send out the package and there are instructions, everything is there for the FIT kit test. And that particular team does what we used to do which is all of the patients that receive this in the mail, they contact them. They contact them, they offer any assistance. They say hey did you get it? Do you need me to send it again? 112

Patients:

- So January of 2021 maybe February, I'll say first quarter as part of the grant we are required to have a patient navigator, we call it registry team that will help those patients who have an abnormal FIT kit, get navigated if you will to the next step, colonoscopy. 102

Telehealth

Description:

- Our medical director of quality, our new medical director has said it's here to stay. She did feel like we were kind of overusing it. She said it shouldn't cap more than 20% of it. And we were up to 40% at one point. So it shouldn't replace in person obviously but we also should be valued the way we were using it. And obviously we have a large subset of diabetic patients and so for some of their metrics they will never be able to get fulfilled via tele-health, like glucose testing, blood pressure screening. 101
- And actually I know they're doing some work now for remote, self-reported screening for glucose or weight or blood pressure. But I think HEDIS changed maybe last year to be able to allow that to meet the requirement. 101
- During the vaccine phase were still doing 60-70% tele-health in each of the clinics, which we have about 20 clinics at that time. And then 30% face to face. So most providers were still working from home at that time. 115
- It became more like 40% tele-health from 60% to 60% face to face. That's happening and we've been able to maintain that now since then. It's been driven more by patient preference.105

Barriers:

- For the registry, tele-health like I said we have great availability at FQHC but the specialists don't. They don't do tele-health. I am not sure if they get reimbursed the same way as if they see patients in person. But this is just me speculating, I am not sure if that's the reason but they don't do it. There's only one doctor I encountered this year that does offer it. So only one of all the GIs in OC and LA county. 110
- Do you find attending that visit in person is a barrier for patients? For a lot of them, it's a lot of hardship because even though we help them with transportation, sometimes they're still unsure. Like they want to go with somebody, they don't want to go alone. Sometimes they have... a little bit of memory problems. I spoke to a patient and she said I forget everything. And I just don't know how I am going to go to this appointment and no one can go with her. 110
- It's very much an option. The other thing that impacted, most of the states, a lot of states let the audio only visit reimbursement expire. It was emergency use authorization is how we were able to get it done. California has one more year this year as a reprieve so that way we can try to develop better video capabilities with our patients. I: How is that going? Does that seem feasible?
  R: No, not really. The issue is not so much on our end it's on their end. We're talking about an under-served population of patients which is our clientele. These are true social determinate barriers that they have. And those are not going to get fixed overnight.105

Facilitators:

- A lot more patients preferred telephone visits. Especially the ones that had to come back for results or the ones that did fecal test came back abnormal and they needed an appointment to have a referral placed. Instead of them physically coming into the office, they would just call them over the phone, like a consult and let them know your test came back abnormal. We're going to refer you out to gastro, and you should receive a referral in the mail within a week or two. A lot of the patients were grateful for that since they didn't have to go all the way into the clinic for that reason. 107
- So a week before the colonoscopy, I call them. I call them using Webex, which is kind of like what we're using now. I try to call as many as possible. Sometimes I have 5 patients, I don't think I've ever had more than 6. I talk with them about how to prepare. Give them a little bit of a background, what is a colonoscopy, why you need it, what happens during a colonoscopy, how polyps are removed, what the doctor does. It's super easy to understand information. And before the class we actually mail them the presentation. So the same exact thing I am going to talk to them we mail it to them so they can follow along or they can read it before and if they have [Vendor]ions they can have those [Vendor]ions fresh in their mind. And then you help them engage with each other a little bit. They have [Vendor]ions and I feel like those [Vendor]ions can help everybody. I think... I believe that of all the patients I have called and give them the bowel prep class, I think only 3 patients didn't have a good bowel prep.
  At thew beginning we wanted to do it that way. We wanted to do more of visual type of thing where they can actually see my face. We encountered that a lot of patients don't know how to use the camera. Or they were flustered with logging in and trying to see me and trying to see the slides. Some couldn't see because their phone was too small. So then we said you know what, we're just going to mail them the presentation and we're going to call them. It's going to be like a phone call. But instead of calling one we're going to call five at the time.

> Whoever signs up because we call them ahead of time and ask them do you want to do... We do Wednesdays for the English speakers and we do Thursday for the Spanish speakers. And we do either 10:00 AM or 5:00 PM. So we ask them which one would you like to attend. 110

Current/now (summer 2022)

Current state description (on clinic, patients, general care, and cancer screenings)

- I feel like we'll never be able to catch up. Especially because the FIT tests are repeated yearly. Technically at the beginning of this year we had the ability to almost reset for FIT tests. But if we're still not going to get as many to come in in person, the delivery will be difficult, we need more resources. But also understanding that we'll never meet the goal with just FIT tests alone. We need a sizable chunk of colonoscopies which have a longer term compliance. And if we can barely get patients in for abnormal colonoscopy, I just have a theory that's not happening for screening colonoscopies. 101
- A lot of the times when the back office, the provider would receive an abnormal result they would send it to their provider partner would receive an abnormal result. They would send it to their provider partner, which is the MA that is assigned to them. That provider partner would have to reach out to the patient and let them know of everything else. But at that time there were overwhelmed with a lot of other things. So by the time the back office would get to the message it could be days or weeks. So now with the registry since that's all we're focusing on, the provider, it feel like it's easier for them to send us the message and it takes the workload off the back office as well. 107
- And we're focusing on reaching out to the patients and explaining to them the results and the next step for them to see the specialist for a consult that they most likely will get a colonoscopy. And we explain to them, if they have any [Vendor]ions about the procedure of how to take the bowel prep medication, if they need transportation, all that we help them with it. So I feel it's easier and it's better for not just the staff but the patient as well. 107
- We're just about back up to speed pre-pandemic. We've resumed our outreach services, meaning our quality team has focused on these measures. Sent out text messages, robo calls, ramped up our outreach and the way that we communicate to the patients to notify them of the need of their screenings so we have that. We've actually seen some work in our quality data which we do have, that there has been a great improvement in a lot of the work around our screening patients are coming for their appointments. Ride share services are up and running. We've had success in getting patients in the clinic. There's been a designated focus particularly around colorectal screenings as of late, making sure that Fit kits were available. 108
- I would say patients don't have any problems in terms of exposure. Like they're not worried about going to their doctor and possibly getting exposed to Covid or anything like that. What I see now is that a lot of the GI's are impacted. They don't have appointments available for 3, 4, 5, 6 months. 110
- I have to keep asking the referrals department to redirect their referrals to another office. And sometimes I have spent hours calling GI offices to ask what's your availability because they don't have anything available. A lot of patients get a little discouraged because my appointment is going to be in 4 months and what do I do? 2021 I was able to get appointments in two weeks, three weeks max. And now it seems that it's getting harder. And some GI offices are not even answering the phone anymore. It just goes straight to VM. 110
- Referral impact in 2021 it was maybe 6-8 weeks. Now we're trying to be 4 weeks. 115
- At that point I had a whole front office team, so I was able to designate certain times and days for every single person to do outreach. And then as far as the CRC screenings, definitely continuing to mail them out. I see a lot of our back office mailing them out. I see a lot more patients bringing them in. So we're turning to not normal, but a new normal, a post-Covid normal. 118
- FQHC created a registry team so there's a colorectal cancer registry team, a cervical cancer registry team and I want to say there's also a mammo registry team. So taking that work off of the clinic staff because now we've been so impacted with just patients wanting to see their providers for anything and everything. 118
- And then the colon cancer screening, I think our FIT campaign last year was very helpful, because it got some... of those... I think our results would've been much worse if we hadn't done it. 105
- Just to throw a wrench in all of this... our denominator increased by 15,000 patients in a month because we now added the new age group of 45-49 for colon cancer screening. So again, our population, the cohort is up by 15000 more patients so there's a lot of work to be done there. 105

S3 Appendix: Minimal anonymized qualitative data set

- Now we have a new Medical Director of Quality, she's amazing. She's been with us for a year. I would say we have spent an incredible amount of time working on making sure our Epic care gaps are easy to, are firing appropriately, easy to see and link to the last test quickly for our providers. And also that our physicians are aware of what are the clinical guidelines triggering those care gaps for the cancer screenings. We have focused a ton of time on training and monitoring on where to go to look at what patients are due at the panel, at the clinic level, with our clinic directors and site medical directors and also with the SDMs. With colorectal we've… we recently started working with having Cologuard instead of FIT kits for our patients for screening. 116
- We started that in late 2021, early 2022. We kind of put our quality outreach and our quality list on the back burner for quite a while. We were still always held accountable to it. We still received month updates on it. But our numbers all plummeted obviously because we weren't doing the preventative, and seeing a lot of patients in clinic. You can't obviously do a bunch of cancer screenings over the phone. So that kind of started really picking up probably the beginning of 2022. We're back to normal speed now….We still were struggling to get patients in at the end of 2021. By the beginning of 2022 patients were really starting to pick up with re[Vendor]ing to come in for physicals. Or saying hey I think I have a problem, I've had it for a year. I really think it needs to get checked, so it really started ramping up in probably January of 2022. But to where we really hit that this feels pretty normal again was probably by March of 2022. 2021, there was a lot of hesitancy especially with our older population. They did not want to come into the clinic still. They were very much afraid because we would have these surges and pockets. And then with Omicron came it went poof and died off again. [garbled] When Omicron hit that put us backwards or stalled us out more than anything, it kind of just stalled us. 114

Ongoing barriers to cancer screening:

- We have patients who test positive and then have to reschedule their colonoscopy. 101
- I don't want to do the prep. I didn't like the provider, this is after they attend the consult. I can't take time off work. High co-pay. I am scared it's going to hurt. Having to show the validity of the initial abnormal test. Yeah. 101
- Lots of Fit kits going around, kind of drive me crazy. We're really looking at how do we shift from this constant churning of FIT kits and really get patients just to do the colonoscopy. And we're also looking at incorporating Cologuard for a 3 year test as well. Colorectal cancer is handing out FIT kits, that's really our push right now. 102
- We're finding FIT kits aren't always the best thing. They're handed out, we have patients who have had abnormal FIT kits and they just keep getting a FIT kit every year. 102
- We know we have a challenge with patients who are uninsured or under-insured getting them to that next colonoscopy. 102
- FQHC has increased our membership by almost 15 – 20,000 patients in the last year, so a lot of our cancer measures have been seriously impacted as well by that. With difficulties of having patients come back in for their cancer screenings or any healthcare in general, we've faced a lot of challenges to... having those patients come in. But for Fit kits and paps we did identify that in general CRC screening nationally has been impacted by about 40%. So even for us having our patients come in to return a Fit kit or even as we're moving towards trying to tackle Cologuard just having patients come and do that has been incredibly difficult. 115
- Even me, I come from a Hispanic household, so they say don't get your mammo because it's going to cause cancer. Don't get testing you don't need to do it now. That has always been there, but now it's more everything. Every preventative measure, what happens if I go here, what are they putting in my body. What is being placed... it's more conspiracy theories and just really educating our patients to understand that there's a difference between what happened and your preventative care. 112
- I: *Maybe you could talk about how things are now in July of 2022?* R:It's much better. The patients like I said they are coming, we are calling. We get the kits, we get the list of whose missing their HEDIS. So we are doing outreach. Our front office team does outreach 3 days a week. There's somebody dedicated to doing outreach 3 days a week. So they'll call our list and our quality team provides us those lists for who is missing. So that's how we're trying to get them all scheduled. And like I said we instituted for June and July we had very specific pap days, where we just did paps with a certain provider. That would be 20 paps a day that she was doing so that helped. *I: Have you recently or are you planning any... promotional things for either breast cancer screening or colorectal cancer screening?...* Some of the health plans offer gift cards if the patient comes in and gets one and can prove it and we give them a form to fill out. Cal Optima, they can send those in and they'll earn $25 gift cards if they come in and complete cervical cancer or breast cancer or CRC. So the staff provides those fliers to the patients that says hey if you come in and get this you know you'll get a gift card. In addition to that when they're not doing that program we offer gift cards as

well on different months when that's our promotion month. And if they come in then we give them a gift card for completing if they come in and bring their FIT kit back we hand them a Walmart or Target gift card. 114
- *I: [2021 to now] You were saying you used to be able to do these tabling events at the clinics where you might be able to talk about encouraging people to get their breast cancer screening or their cervical cancer screening or to complete their FIT test. It sounds like once the pandemic hit and you guys went virtual you haven't been doing any kind of that education?* R: To be honest we don't really focus on cancer screenings, other than we mention it on one of our diabetes group classes which is the main one we do focus on. We do mention it, it's on week 2. Because it's a 6 week program we talk about cholesterol and hypertension and then we talk about making sure they're getting their lab work done. And at the very end we add to make sure they're also getting their cancer screenings. Honestly that's all the mentioning we do at the moment. 117

Improvements or future plans for cancer screenings:

- I think for colorectal, the inclusion of Cologuard and fixing the data will give us a better picture of really where we're at. And I'd also like to see about... again when we get more access to specialists in the gastro category, I'd like to see about how we can drive patients to getting colonoscopies rather than the constant FIT kit churn. 102
- In the past we had discussions about Cologuard and now that's back on the table again. So it looks like it's going to happen this time. So that's a nice option and it's got better in my view, it's kind of the midway between specificity and sensitivity and all that, it's better. It's not a colonoscopy but it's better than FOB and it gets you a 3 year screening time. 104
- Again I would… I think you have to have a tiered approach in terms of identifying where patients are. I don't know if you ever heard [Name] and others do this, there's different flavors of customer. The dog is the king or whatever and they have an approach for each of those type of people. I am not saying to label people, but you can bucket them to understand there's got to be common themes. No that's gross I don't want to deal with it, how do you deal with that? And I don't know maybe there was some sort of trauma that people don't want to deal with colonoscopy. I think that would be cool. 104
- I can't argue enough, I think we give lip service to evidence-based medicine. And I think, I look forward to the day where we can really be able to have discussions with people about risk benefit. I really don't see that happening. And we do shadow providers, and they work to watch them use the EMR. But people don't have discussions, screen, well there's no discussion about it could be false positive, it could be false negative. That kind of stuff, I would love to see us do better at. 104
- More patient education. More things that catch the eye and that they're aware of. Like you'll hear in the news, XY&Z passed away because they had colon cancer and that's the end of that. There's no follow up. It's important to get this done. And at this age... or anything educational, whether it's a commercial. I've seen FQHC that have commercials about scheduling appointments and all that and having their health checked. But I feel like it should be focusing on in general little things, like okay women pap and mammo, both genders do colorectal screening. If you're diabetic you need to get things done. It shouldn't be a general thing, it should be little things that should be focusing on so everyone is aware of everything. 107
- I think the problem is they need a lot of things to be approved from marketing and stuff. But the little telenovela of how FQHC made the video, it's on YouTube. It gives them a scenario and explains how the exam is done. I saw the video and I am like this is awesome. 107
- I think the overall how we do care, the inclusion of tele-health, the creativity and flexibility to be able to mail out Fit kits and have a process developed around there to where patients can just come in and drop off. I think that's not going anywhere. 108
- So, I actually run out outreach program, at least for the HEDIS measures. So historically we used to do a mass Fit kit mailer, tackling 30,000 Fit kits, and mailing them manually to all these patients and it was a very fruitless effort. Even though we see an uptake of about 6% across FQHC it wasn't quite making the numbers, nor was it... beneficial for how much we were paying for it. So, this year I've been doing a monthly opt in mailer. So, we do text messages to about 2000 patients a month. If they opt in to receive a Fit kit, we send them that Fit kit and then we do a follow up additionally. That's increased our compliance rate of 50%. So, of about 5000 Fit kits we've sent out this year we've received 2500 back. 115
- I was definitely hoping to collaborate with FQHC in the future to try to do a lot of community outreach to our patients, to encourage them and remind them that many of them feel like they're family at FQHC and just wanted to

let them know their family is still there for them too. We just want them to be healthy and that's our ultimate goal. 115

- I can probably make a list, but definitely staffing. I think we definitely need to be able to expand our services more. I know FQHC is also working to get a GI specialist in one of the clinics so that would even help abnormal Fit kits. Other things is a round the clock staffing for our call center. I know that's really hard but I hope that these changes and adjustments to the salaries will encourage more people to come and work for the call center. Same with our outreach programs. Our outreach program is ultimately affected by how many people are staffed at our call center. The less people that are staffed there, the slower our outreach happens. 115
- Our staff doesn't have so much time to follow up on abnormal results. So taking that out of the clinic and having specific teams dedicated to when they get the abnormal call and the patient scheduled, scheduling with a specialist... Doing all of that work for us, I think that was a really great idea and a great success. 118
- The thing, I think with the FIT Kits that's the struggle that we've had and I think we have room for improvement, just patient education, sharing videos. We still get those [Vendor]ions of how do I do this, I don't know how to do it, I don't know if I did it right. 118
- For the colorectal that's something the patient has to do by themselves. And sometimes I think it can be intimidating for our patient population to do. So I think that's probably the one area that I would really like to see improve. And I know this year they also expanded the age ranges so we have a lot more patients we have to cover for that. So I would really like to see us improve our patient education of that so that they can feel comfortable and confident doing it. I think that's a definitely a better solution than sending them to get their colonoscopies, just because our patient population it's difficult to get them to schedule with specialists. 118
- So we're doing now continued outreach and we're doing it in a focus, we found the focused method a little bit better. But we're finding new barriers to that as such. And then also the GI offices are back open so we're able to now, start getting patients in. But we're also exploring other options. We're looking at Cologuard now. I have a meeting next week to sort that out. It's very hard to do colon cancer screening every year for 70,000 patients. 105
- There are still definitely the same barriers we're seeing with telehealth. We have about 32% of our patient population that signs into our portal. So we've been really pushing for that over the last year and a half and we're revamping our portal as we speak in in order to make it more user friendly and provide more options for patients like being able to pay their bill through the app, and previously there was a check in option but it didn't quite work where we could see the patient had actually checked in. So we're just making all those things much more user friendly for the patient. 111
- I think it all rests in education. If we can do a better job of educating our patients on the importance of it there's a lot of misinformation and misperceptions of what they are and how useful they are. And if we can leverage more technology or social media, or something, multiple modalities to reach our patients and talk to them about the importance. I know we're doing it but I just hope in time more patients are getting the correct information as opposed to relying on social media from Joe Schmo that says you don't want to do this because of XYZ and has no medical facts to it. 111
- Honestly, I think in our clinics is where we really see it the most with our providers. It's more of what we see our providers do. I don't know how they do it because their slots are so limited. It's 20 minutes and they get double-booked. How do we give them more time with the patients to give them that education piece. I think and I am no provider but they look at what's in front of them. But when do we start talking about the fear behind certain services that the patients have. 112
- As far as the material itself, we do. We have our communication team that consistently sends out communication. We even have a Covid support line within Alta-Med and we advertise that. If you need any resources and they have a huge thing, a lot of resources. Housing, financial, really what Covid impacted you, looking for employment. Just talking to somebody, getting those coping skills in. So we have that team that works on that. And that was wonderful to see because now we're starting to add a little bit more of that educational piece. 112
- I: *Do you think it's easier to motivate people to follow through on something if it's a face to face conversation versus a tele-health visit too? If you're trying to overcome resistance to FIT screening?* R: 100%, 100%. Even to touch a patient to have that... passion and compassion and empathy when I can sit in front of a patient and when our providers sit in front of them, patients are a lot more receptive and expressive when they're in front of you than they are over the phone. Just like the rest of us there's stuff going on. And I hate doing... our offices, we've got to be in person at some point. This is crazy, we're missing nuances, we're missing things when we're not physically in each other's presence. And I think with patients, they've got extraneous noises, especially the mommies. They've got kids

running around, it's so quick and rushed and I don't feel like they disclose the way they would disclose if they were with us in person. I think it's a barrier to do video visits sometimes. And older population honestly can't navigate it. They can't, it's very difficult for them and how many patients have we missed because they couldn't navigate that video visit and so they no showed. Our no show rates for video visits was high for a while. It was up to 25 percentile, where a quarter of the patients just couldn't do it. They couldn't navigate the phone, they couldn't navigate the video. So were we giving substandard care? I don't know. I'll be interested when all of this finally goes and people really start doing the deep dive and researching, did our cancer rates go up, did our death rates go up, what happened? … we tell stories. Obviously we don't use specifics but we tell them hey things are getting missed. If we catch you at Stage 1, same as I said, versus we don't catch you until Stage 4 the outcome, treatment, the prognosis is much different. And we tell them when we call them we have not seen you in two years. It's imperative that you come in, that we examine you and check you and do all your screening. A lot can change in two years. We need to see you physically, we need you to come back, for your own health and well being. So I think we change our scripting based on what the current climate is anyway. FQHC is really good about, okay we've all tried this, we've done it for 6 months, it's not showing a great return, let's come up with something different. And we all share best practices. We go to the clinics that have the best FIT kit return and say what are you doing that maybe we're not doing so we can try and see if it works. And not all the same things work at all our same clinics. We all have different populations. And we have to temper our approach to our particular clinic's population. 114

- I said my passion and my impetus is to get these providers back into the offices more so that we can get these patients seen. One we need more providers, two they need to be here. We need to see our patients face to face. A few telephone calls a day embedded into schedules, great, for those easy things oh hey I have a UTI. I know it's a UTI, okay you need an antibiotic. Do we need to see that patient face to face? Probably not. If I looked at their Epic and see they have not done any of their HEDIS to date then I would say I will see you for this UTI but I am also going to schedule you because you have not had a pap. You have not had your mammo. You have not done a fit test. You don't have your flu shot, let's get you in, because we need to see you for all these other things. I think that should be the push any time that we do a telephone visit now. It should be that's great. I am going to handle this right here, right now. But as I get you off the phone I need to schedule you for XYZ. 114
- I would say we still have the same opportunities to improve in the areas that we did before. But I also think having the telehealth component is such a huge win for us because it provides another modality that makes it a lot easier for our patients to access us and for us to access them. Including the nudging that that can take sometimes if you don't want to do one of those screenings and you need to. I will say changing CRC guidelines for 45 and up is a much heavier lift. It's a lot more patients, so that has definitely been problematic. But I think having cologuard as an option for us has been huge because at least it's good for 3 years. It's an easier sell to the patients too. Exactly. And we're also looking at how we can partner with procedure centers so we can directly refer for colonoscopies. *I: What do you think might facilitate or get in the way of making that happen? Or is it too early to tell?* R: It's too early to tell. We're beginning those conversations. The Medical Director of Quality and I are beginning those conversations now. Sometimes it is not necessarily the contract, it's usually a lot of the technical side that can take us longer. You got to build pipelines for our EMR to talk to the other EMRs so that orders can go through and all that. If Covid taught us one thing it's that we can do things a lot faster than we think we can when we don't have competing priorities. 116
- *I: What about GI, have things improved in terms of getting people into a GI specialist now?* R: Yeah I think with GI the same. They opened a little bit more of their doors for appointment availability. The only thing is they were having issue with was more insurance-wise. If the insurance will cover it, or if the insurance was not covering it. What is the next step that we can do for the patients. 119
- *I: Was that something that existed before Covid? Or something that got worse because of the pandemic, the insurance thing you were just describing?* R: It's always been an issue with insurances. We've always had issues where insurance won't cover the colonoscopy because it's not medically necessary. Obviously it is, because this is a preventative for them. We don't want them to find out God forbid that they have any cells that are active, that's going to cause them to have colorectal cancer. 119
- Before we had this program which was the [?] which was more for patients that didn't have insurance so they were able to get free services to see the GI. But at the same time it was always an issue when it came to appointments and the time. They were always giving them an appointment months from now. 119
- I would say probably once we emerged out of the URI flu pandemic surge that we got in late 2021, as we emerged out of that in early spring 2022, that was when we were able to shift back to much more of a preventative based approach. A couple factors associated with that. One is... I think we had had enough experience with the ups and downs, the peaks and valleys of the pandemic that we were able to quick flex back to surges. We actually do have a

3 stage approach to our surges, 4 stage, 0, 1, 2 and 3. 3 being full surge, zero being no surge whatsoever or basically pre-pandemic levels and 1 being low risk. Once we had gotten back to a level 1, where we were confident we had the staffing, we were confident we had the space. We were confident we had the capacity to manage schedules and all the care coordination and all of that in spring 2021, that was probably when we went back to almost as close to what I would call a new normal. And really started to focus again on really drilling into our clinical processes and our quality based processes, our evidence processes for screening guidelines. We reactivated all of our traditional dashboards, and started looking at all of our incentives and doing our internal communications and working with our front office and back office staff and making sure we know who our call lists were and our outreach plans and all of that. I would say early 2022 is when we really started to bolt into that and get back into some kind of new normal…The biggest crisis was the staffing. By this time we had so much turnover with nursing. A lot of internal promotions. A lot of our supervisors and managers who'd been seasoned line staff with us but new to management. So the idea of them understanding how to do management or oversight of process as opposed to directly delivering care. A lot of that of course because we still could not do all of our in person trainings, we were limited by our own internal space. So you could sense that there was probably some level of erosion of the quality of inefficiency of how well we could do it, just because of how many new staff or how many new managers and all of that. …But in the sense of at least us being able to focus on all of our standard screenings, all of our standard work processes it was there. But the burnout was definitely a huge issue and our continuing all the way through summer and into fall of 2022, the turnover and then the competition with being able to retain. You know the organization did an incredible job from spring through early fall 2022, reassessing the entire competitive pay environment to see what adjustments we needed to make both for existing staff as well as to compete for bringing staff one. So those were all definitely factors. …As an example, we've always been a very people focused organization, or a warm and friendly organizational work culture. And the fact that we just started reinstating in person new hire orientations again these last few weeks. The fact that you're finally starting to get a little bit of that bonding and normalcy back to just feeling like people at least know who we are and who they work for. And just onsite on the job training. 120

Long-term COVID impacts/greatest impacts:

- I think for patients who had an abnormal, and never followed up on possible cancer detection right, definitely polyp existence that's not going to be detected any time soon if they don't follow up. Delay in polyp removal. So I feel like it's going to be GI long term problems if not cancer then other things. 101
- I believe the main thing is job loss which that trickles down to no insurance, having no money to pay for their exams. Focusing on finding a job to help pay for their day-to-day living instead of them focusing on their health. 107
- I think food insecurities continues to be a major concern. Just in general for our patient population but Covid truly added the importance of it or highlighted how many of our patients really do have food insecurities that we may not have known, may not have ever known. And then the need for social determinates and the increase in benefits of mental health. 108
- For patients that do not have medical insurance, and not having a job, that was the main one. But sometimes patients, even if they do have a job they still don't want to pay. Our cheapest colonoscopy is $600, it's still a lot of money. Yeah that's what I hear the most, not being able to pay for it. Because the rest we can work around it but yeah. 110
- It's also definitely our scheduling and our access. We have a 3-month scheduling template so we can schedule for 3 months out. Currently we're now doing 6 months just because of how limited our access is. Even though we're down providers, we don't have enough pediatric providers. We don't have enough women's health providers. Our nurses do also support how many patients are now members within our organization or in our denominator. Usually an example, breast cancer we're now schedule 2-3 months out for their breast cancer screenings. 115
- As far as that long term, I think I spoke to it before, just really changing the culture of the patients. I know there's a lot of SDH that do impact our patient's ability to come into the clinic to have services done. That's also why we have our housing clinics and our traveling providers as well. 115
- I think definitely access has declined greatly in the last couple of years, post Covid. Our providers, they're slowly coming back to face to face. But we've just had so many patients waiting to see their doctor, that now at this point patients are coming in for things. And we tend to see sicker patients, patients with more chronic conditions, just because it hasn't been treated in two years. 118
- It's definitely had an impact, there are people who lost jobs, real reality. They've definitely lost jobs, they're unemployed. Some of them have been afraid to work so they've stayed home and have had financial implications as

well. There's definitely been truly more anxiety, that and depression and isolation. That has definitely been something we have seen. And then, so that's affected many things like housing and food insecurity. So I do think that there has been a big financial impact, yes I do believe that in many sectors which is affecting our patients.105

- The only thing I think I would say I think we will see more of the aftermath of this a little bit later because of the delayed screenings. That's my concern, I don't know obviously for a fact, but because we delayed screenings, of course some patients didn't even get them done, that I think there was this impact from the pandemic and we may be seeing worsening of the rates of colon cancer or breast cancer, more advanced stages because we were late. I think that's my concern.105
- I think overall it's been a positive impact, in that people have now become comfortable with multiple modalities to engage in their healthcare. And that it doesn't require just showing up to the clinic, there's other options to make sure they get the care that they need. 111
- I just think about the overall impact. It's changed the landscape for medicine. There's a lot more distrust in the medical community. There was a lot of vocalization of people's opinions based on whether they thought the vaccines were just to make money or if it was actually true. This is worth for your health than taking a chance of getting Covid. So I think that's been one of the biggest challenges that's going to take a long time to level out. 111
- The challenge of overcoming the fear of Covid is one we will continue to see. Especially with our patient demographic, we have a lot of... well if I go get this screening is it going to cause this because I got vaccinated. It's fear that there's consequences of now I got the vaccine, what happens here. How do you know I am not going to get sick. And getting our patients to really become educated on Covid-19 and then trying to compartmentalize that and not let it affect their preventative measures. I want to say that's the hugest challenge. 112
- 100% we've missed stuff, and it breaks my heart to know it. Because anecdotally I know patients that now we have cancer diagnoses for. I am just thinking had you guys come in we would've caught it sooner. But there was a lot of patients we couldn't get in. They wouldn't come, we didn't have all the access. It was just the perfect storm, being the perfect nightmare basically. My own father anecdotally died from liver cancer, and it was missed because of Covid because he couldn't get in. He just passed in October and I 100% it's because it got missed because he couldn't get in ... Now the pendulum swung the other way and now they're angry that they can't get in sooner and that we don't have as many appointments and that it's taking too long. Our grievances have now swung to oh my God I am trying to get in, how come I can't get in? Why are you telling me it's going to take me 3-4 weeks to get an appointment with my provider. So yeah, it now swings the opposite way, they want to be here now, and they want to be seen. 114

Impact of External Factors (or not)

Disaster plan:

- Played a huge role, we met every week to assess surge planning, to assess workflows. We met with our infectious disease team, infectious disease providers, access and operation leaders and we adjusted. We would look at what was happening in the East Coast, where we trailed, what was the surge doing, what was Covid doing? We would make adjustments to those plans. We could only see 80% of our patients via tele-health this week. We might be able to see 60% of our patients via tele-health depending on where we were in surge planning. We made adjustments to how we did care based upon the recommendations from this team. 108
